# Availability of Evidence for Predictive Machine Learning Algorithms in Primary Care: A Systematic Review

**DOI:** 10.1001/jamanetworkopen.2024.32990

**Published:** 2024-09-12

**Authors:** Margot M. Rakers, Marieke M. van Buchem, Sergej Kucenko, Anne de Hond, Ilse Kant, Maarten van Smeden, Karel G. M. Moons, Artuur M. Leeuwenberg, Niels Chavannes, María Villalobos-Quesada, Hendrikus J. A. van Os

**Affiliations:** 1 Department of Public Health and Primary Care, Leiden University Medical Centre, ZA Leiden, the Netherlands; 2 National eHealth Living Lab, Leiden University Medical Centre, ZA Leiden, the Netherlands; 3 Department of Information Technology and Digital Innovation, Leiden University Medical Center, ZA Leiden, the Netherlands; 4 Hamburg University of Applied Sciences, Department of Health Sciences, Ulmenliet 20, Hamburg, Germany; 5 Department of Digital Health, University Medical Center Utrecht, Utrecht University, Universiteitsweg 100, CG Utrecht, the Netherlands; 6 Julius Center for Health Sciences and Primary Care, University Medical Center Utrecht, Utrecht University, Universiteitsweg 100, CG Utrecht, the Netherlands

## Abstract

**Importance:**

The aging and multimorbid population and health personnel shortages pose a substantial burden on primary health care. While predictive machine learning (ML) algorithms have the potential to address these challenges, concerns include transparency and insufficient reporting of model validation and effectiveness of the implementation in the clinical workflow.

**Objectives:**

To systematically identify predictive ML algorithms implemented in primary care from peer-reviewed literature and US Food and Drug Administration (FDA) and Conformité Européene (CE) registration databases and to ascertain the public availability of evidence, including peer-reviewed literature, gray literature, and technical reports across the artificial intelligence (AI) life cycle.

**Evidence Review:**

PubMed, Embase, Web of Science, Cochrane Library, Emcare, Academic Search Premier, IEEE Xplore, ACM Digital Library, MathSciNet, AAAI.org (Association for the Advancement of Artificial Intelligence), arXiv, Epistemonikos, PsycINFO, and Google Scholar were searched for studies published between January 2000 and July 2023, with search terms that were related to AI, primary care, and implementation. The search extended to CE-marked or FDA-approved predictive ML algorithms obtained from relevant registration databases. Three reviewers gathered subsequent evidence involving strategies such as product searches, exploration of references, manufacturer website visits, and direct inquiries to authors and product owners. The extent to which the evidence for each predictive ML algorithm aligned with the Dutch AI predictive algorithm (AIPA) guideline requirements was assessed per AI life cycle phase, producing evidence availability scores.

**Findings:**

The systematic search identified 43 predictive ML algorithms, of which 25 were commercially available and CE-marked or FDA-approved. The predictive ML algorithms spanned multiple clinical domains, but most (27 [63%]) focused on cardiovascular diseases and diabetes. Most (35 [81%]) were published within the past 5 years. The availability of evidence varied across different phases of the predictive ML algorithm life cycle, with evidence being reported the least for phase 1 (preparation) and phase 5 (impact assessment) (19% and 30%, respectively). Twelve (28%) predictive ML algorithms achieved approximately half of their maximum individual evidence availability score. Overall, predictive ML algorithms from peer-reviewed literature showed higher evidence availability compared with those from FDA-approved or CE-marked databases (45% vs 29%).

**Conclusions and Relevance:**

The findings indicate an urgent need to improve the availability of evidence regarding the predictive ML algorithms’ quality criteria. Adopting the Dutch AIPA guideline could facilitate transparent and consistent reporting of the quality criteria that could foster trust among end users and facilitating large-scale implementation.

## Key Points

**Question:**

Which machine learning (ML) predictive algorithms have been implemented in primary care, and what evidence is publicly available for supporting their quality?

**Findings:**

In this systematic review of 43 predictive ML algorithms in primary care from scientific literature and the registration databases of the US Food and Drug Administration and Conformité Européene, there was limited publicly available evidence across all artificial intelligence life cycle phases from development to implementation. While the development phase (phase 2) was most frequently reported, most predictive ML algorithms did not meet half of the predefined requirements of the Dutch artificial intelligence predictive algorithm guideline.

**Meaning:**

Findings of this study underscore the urgent need to facilitate transparent and consistent reporting of the quality criteria in literature, which could build trust among end users and facilitate large-scale implementation.

## Teaser

This systematic review assesses the quality of evidence from scientific literature and registration databases for machine learning algorithms implemented in primary care to predict patient outcomes.

## Introduction

In most high-income countries, primary health care is affected by the increasing burden of illness experienced by aging and multimorbid populations along with personnel shortages.^1^ Primary care generates large amounts of routinely collected coded and free-text clinical data, which can be used by flexible and powerful machine learning (ML) techniques to facilitate early diagnosis, enhance treatment, and prevent adverse effects and outcomes.^2,3,4,5^ Therefore, primary care is a highly interesting domain for implementing predictive ML algorithms in daily clinical practice.^6,7,8^

Nevertheless, scientific literature describes the implementation of artificial intelligence (AI), especially predictive ML algorithms, as limited and far behind other sectors in data-driven technology. Predictive ML algorithms in health care often face criticism regarding the lack of comprehensibility and transparency for health care professionals and patients as well as lack of explainability and interpretability.^8,9,10,11^ Additionally, the reporting of peer-reviewed evidence is limited, and the utility of predictive ML algorithms in clinical workflows is often unclear.^8,12,13,14^ In response to these challenges, the Dutch Ministry of Health, Welfare, and Sports commissioned the development and validation of a Dutch guideline for high-quality diagnostic and prognostic applications of AI in health care. Published in 2022, the Dutch Artificial Intelligence Predictive Algorithm (AIPA) guideline is applicable to predictive ML algorithms.^15,16^ The guideline encourages the collection of data and evidence consistent with the 6 phases and criteria outlined in the AI life cycle (requirements), providing a comprehensive overview of existing research guideline aspects across the AI life cycle.

In this systematic review, we aimed to (1) systematically identify predictive ML algorithms implemented in primary care from peer-reviewed literature and US Food and Drug Administration (FDA) and Conformité Européene (CE) registration databases and (2) ascertain the public availability of evidence, including peer-reviewed literature, gray literature, and technical reports, across the AI life cycles. For this purpose, the Dutch AIPA guideline was adapted into a practical evaluation tool to assess the quality criteria of each predictive ML algorithm.

## Methods

We conducted the systematic review in 2 steps. First, we systematically identified predictive ML algorithms by searching peer-reviewed literature and FDA and CE registration databases. Second, we ascertained the availability of evidence for the identified algorithms across the AI life cycle by systematically searching literature databases and technical reports, examining references in relevant studies, conducting product searches, visiting manufacturer websites, and contacting authors and product owners. We followed the Preferred Reporting Items for Systematic Reviews and Meta-Analyses (PRISMA) reporting guideline.^17^

### Eligibility Criteria

Peer-reviewed studies were included if they met all of the following eligibility criteria: (1) published between January 2000 and July 2023; (2) written in English; (3) published as original results; (4) concerned a predictive ML algorithm intended for primary care; and (5) focused on the implementation of the predictive ML algorithm in a research setting or clinical practice with, for example, pilot, feasibility, implementation, or clinical validation study designs. This review examined ML techniques (eg, [deep] neural networks, support vector machines, and random forests) developed for tasks such as computer vision and natural language processing that generated the prediction of health outcomes in individuals. We classified a study as applying ML if it used a nonregression statistical technique to develop or validate a prediction model, similar to Andaur Navarro et al,^9^ excluding traditional statistical approaches, such as expert systems and decision trees based on expert knowledge. Studies that addressed predictive ML algorithm development or external validation without implementation in primary care were excluded. Given that over 60% of CE-marked AI tools are not found in electronic research databases,^12^ this review included CE-marked or FDA-approved predictive ML algorithms published in FDA and CE registration databases.^18,19,20^

### Data Sources and Search Strategy

Searches were conducted using the following electronic databases: PubMed, Embase, Web of Science, Cochrane Library, Emcare, Academic Search Premier, IEEE Xplore, ACM Digital Library, MathSciNet, AAAI.org (Association for the Advancement of Artificial Intelligence), arXiv, Epistemonikos, PsycINFO, and Google Scholar. All databases were searched in July 2023 for entries from January 2000 to July 2023. The search terms, derived from the National Library of Medicine MeSH (Medical Subject Headings) Tree Structures and the review team’s expertise, formed a combination related to AI, primary care,^21^ and implementation^22^ (defined in Box 1). The full search strategy is provided in eAppendix 1 in Supplement 1.

Box 1. **Definitions**

**Predictive Algorithm**

“An algorithm leading to a prediction of a health outcome in individuals. This includes, but is not limited to, predicting the probability or classification of having (diagnostic or screening predictive algorithm) or developing over time (prognostic or prevention predictive algorithm) desirable or undesirable health outcomes.”^16^

**Implementation**

“An intentional effort designed to change, adapt, or uptake interventions into routines, including pilot and feasibility studies.”^22^ Implemented predictive ML algorithms can be placed in phases 5 and 6 of the AI life cycle, as defined by the Dutch AIPA guideline.^16^

**Primary Care**

“Universal access to essential healthcare in communities, facilitated by practical, scientifically sound, socially acceptable methods and technology, sustainably affordable at all developmental stages, fostering self-reliance and self-determination.”^21^

Abbreviations: AI, artificial intelligence; AIPA, Artificial Intelligence Predictive Algorithm; ML, machine learning.

### Selection Process

Three of us (M.M.R., M.M.vB., and S.K.) conducted an independent review of the selection process, resolving disagreements among us through discussion with a senior reviewer (H.J.A.vO.). The full selection process is detailed in eAppendix 2 in Supplement 1.

### Data Extraction

Five strategies were used to gather publicly available evidence for all identified predictive ML algorithms: (1) searches of PubMed and Google Scholar using product, company, and author names; (2) searches of technical reports from the FDA and CE registration online databases; (3) exploration of references within selected studies; (4) visits to predictive ML algorithm manufacturer websites; and (5) solicitation of information from authors and product owners via email or telephone, with a request to complete an online questionnaire about the reported evidence (eAppendix 3 in Supplement 1). Accepted data sources included original, peer-reviewed articles in English as well as posters, conference papers, and data management plans (DMPs).

The availability of evidence was categorized according to the life cycle phases (Box 2) and the requirements per phase set forth by the Dutch AIPA guideline.^15,16^ These requirements (Table 1) are defined as aspects necessary to address during the AI predictive algorithm life cycle. Therefore, developers, researchers, or owners of predictive ML algorithms should ideally provide data and evidence regarding these aspects.

Box 2. **Summary of the 6 Life Cycle Phases^a^**

**Phase 1: Preparation and Verification of the Data**

A DMP should be used to prepare for the collection and management of the necessary data for phases 2 to 5. In this plan, agreements and established procedures for collecting, processing, and storing data and managing this data are captured. During the implementation of the DMP, any changes should be continuously updated.

**Phase 2: Development of the AI Model**

The development of the AI model, which results from the analysis of the training data, entails the development of the algorithm and the set of algorithm-specific data structures.

**Phase 3: Validation of the AI Model**

The AI model undergoes external validation, which involves evaluating its performance using data not used in phase 2. The validation process assesses the statistical or predictive value of the model and examines issues related to fairness and algorithmic bias.

**Phase 4: Development of the Necessary Software Tool**

The focus shifts to developing the necessary software tool around the AI model. This phase encompasses designing, developing, conducting user testing, and defining the system requirements for the software.

**Phase 5: Impact Assessment of the AI Model in Combination With the Software**

This phase determines the impact or added value of integrating the AI model and software within the intended medical practice or context. It evaluates how these advancements affect medical actions and the health outcomes of the target group, such as patients, clients, or citizens. Additionally, conducting a health technology assessment is part of this phase.

**Phase 6: Implementation and Use of the AI Model With Software in Daily Practice**

The AI model and software are implemented, monitored, and incorporated into daily practice. Efforts are made to ensure smooth integration, continuous monitoring, and appropriate education and training related to their use.

Abbreviations: AI, artificial intelligence; DMP, data management plan.

^a^
As established in the Dutch AIPA guideline.^15,16^

**Table 1.** Overview of Requirements Within the Dutch Artificial Intelligence Predictive Algorithm Guideline for High-Quality Diagnostic and Prognostic Applications of Artificial Intelligence in Health Care

Artificial intelligence life cycle phase	Requirement	Maximum score of availability of evidence per requirement
Phase 1: Preparation	Data management plan	2
Phase 2: Development	Definition target use	14
	Analysis and modeling steps	
	Internal validation	
	Robustness	
	Size of the dataset for AI model development	
	Reproducibility and replicability	
Phase 3: Validation	Evaluation of statistical characteristics of the artificial intelligence model	12
	Fairness and algorithmic bias	
	Determining the outcome variable	
	Reproducibility and replicability	
	Size of the dataset for external validation	
Phase 4: Software application	Explainability, transparency, design, and information	4
	Required standards and regulations	
Phase 5: Impact assessment	Impact assessment	10
	Health technology assessment	
Phase 6: Implementation	Implementation plan	6
	Monitoring	
	Education	
Total availability score	Not applicable	48

### Statistical Analysis

The extent to which the evidence of each predictive ML algorithm aligned with the requirements of the Dutch AIPA guideline was assessed per life cycle phase (Table 1; Box 2; eTable 1 in Supplement 1), using availability scores (2 for complete, 1 for partial, and 0 for none). Two analyses were conducted. First, availability of evidence per requirement was represented as a percentage, considering the requirements per life cycle phase. The availability of evidence per life cycle phase was reported as a percentage and calculated by dividing the sum of scores of a specific life cycle phase by the maximum possible score. Second, evidence availability per predictive ML algorithm was calculated as the sum of values for all requirements divided by the total applicable requirements, excluding the requirements that were not applicable because of the life cycle phase of the algorithm (eTable 1 in Supplement 1; requirements are shaded in orange); the denominator value was 48. These availability scores aimed to provide an overview of implemented predictive ML algorithms and evidence per life cycle phase.

The analysis was conducted independently by 3 of us (M.M.R., M.M.vB., and S.K.), who resolved discrepancies through discussion with another author (H.J.A.vO.). Data were analyzed using Microsoft Excel for Windows 11 (Microsoft Corp).

## Results

### Predictive ML Algorithms Implemented in Primary Care

**Figure 1.**
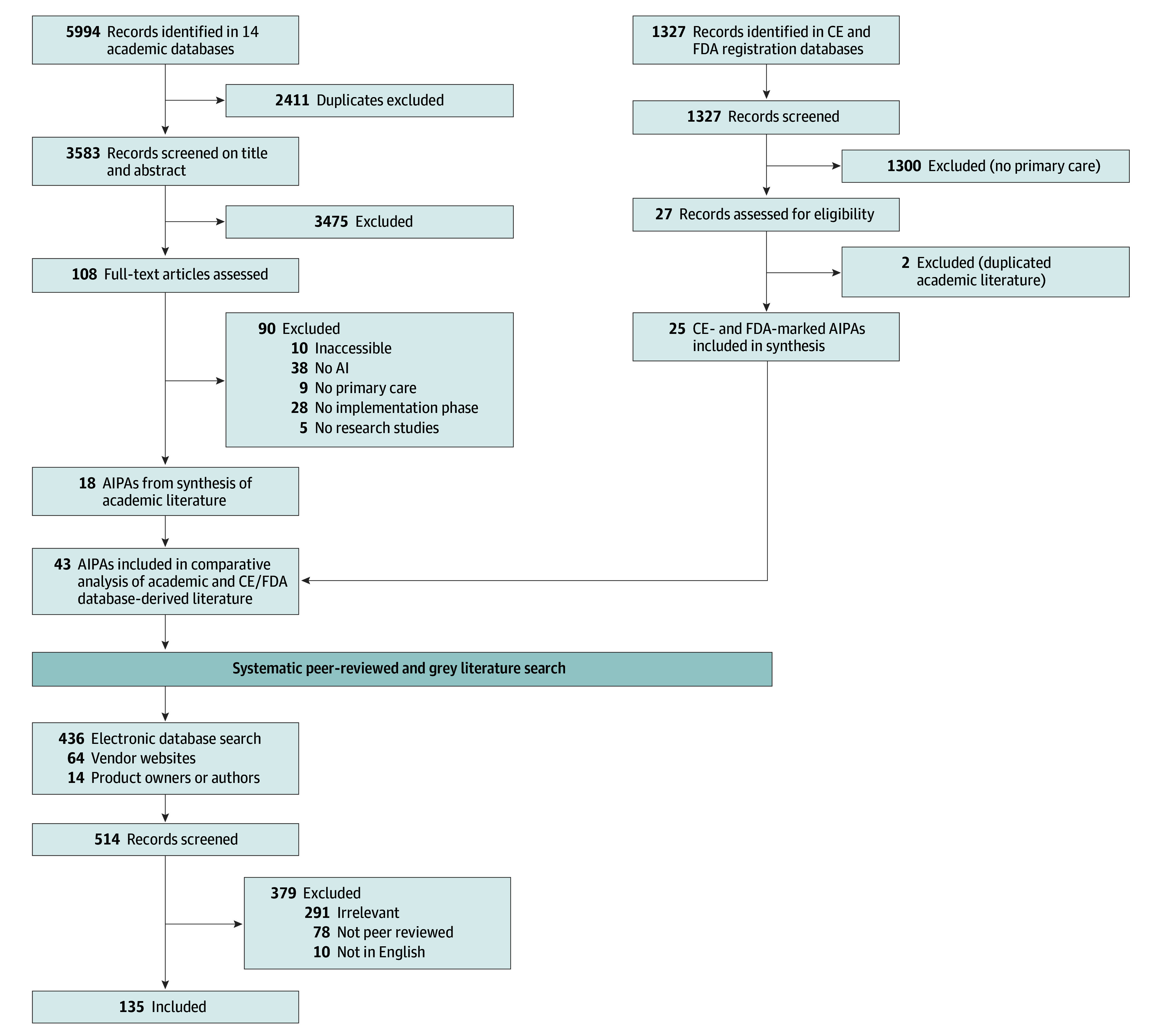
PRISMA Flowchart AI indicates artificial intelligence; AIPA, artificial intelligence predictive algorithm; CE, Conformité Européene; FDA, US Food and Drug Administration.

Of the 5994 studies identified initially, 20 (comprising 19 predictive ML algorithms) met the inclusion criteria and were included in this systematic review. One algorithm was excluded after personal communication confirmed that the tool used was a rule-based expert system.^23^ Additionally, 25 commercially available CE-marked or FDA-approved predictive ML algorithms in primary care were included.^18^ Only 2 predictive ML algorithms were found in the FDA or CE registration databases and the literature databases searched.^24,25,26,27^ Forty-three AIPAs were included in the analysis (Figure 1).^24,25,26,27,28,29,30,31,32,33,34,35,36,37,38,39,40,41,42,43,44,45,46,47,48,49,50,51,52,53,54,55,56,57,58,59,60,61,62,63,64,65^

**Figure 2.**
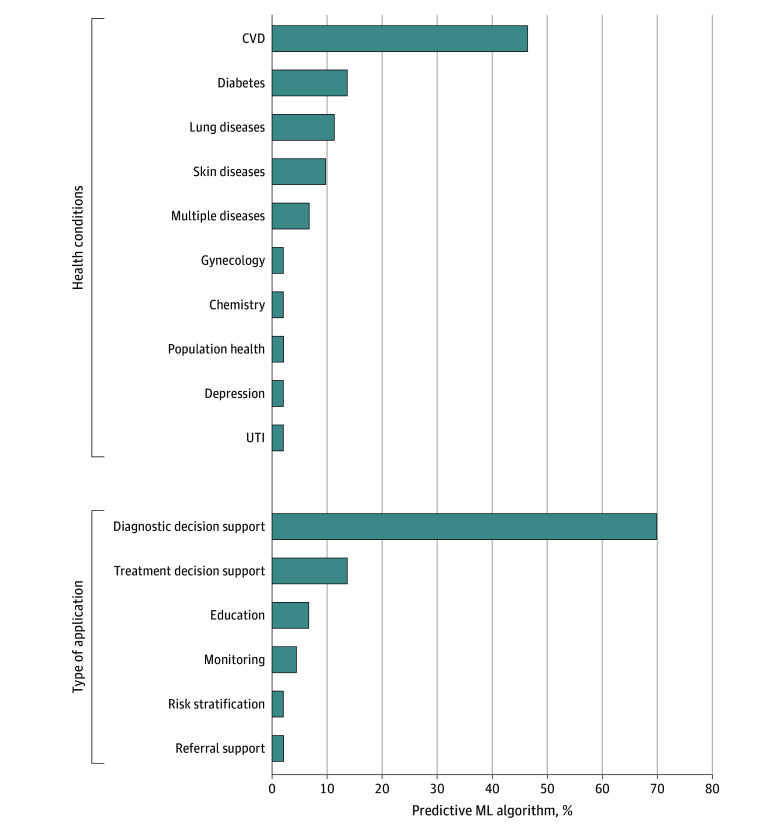
Overview of the Type of Application and Health Conditions Targeted by the Included Predictive Machine Learning (ML) Algorithms CVD indicates cardiovascular disease; UTI, urinary tract infection.

Table 2 provides an overview of the key characteristics of the 43 predictive ML algorithms included in this review.^24,25,26,27,28,29,30,31,32,33,34,35,36,37,38,39,40,41,42,43,44,45,46,47,48,49,50,51,52,53,54,55,56,57,58,59,60,61,62,63,64,65,66,67^ Most studies (35 [81%]) were published in the past 5 years (2018-2023).^24,26,27,28,29,30,31,32,33,34,35,36,40,41,42,43,44,45,46,50,51,52,53,54,55,57,58,59,60,61,62,64,65,66,68^ Most predictive ML algorithms (36 [84%]) fit under the category of clinical decision support systems for either diagnosis or treatment indication (Figure 2).^24,25,26,27,28,29,31,32,33,34,35,37,38,39,40,41,42,44,46,47,49,51,52,53,55,56,57,58,59,60,61,63,64,65,66,67^ Twenty-seven predictive ML algorithms (63%) focused on cardiovascular diseases and diabetes (Figure 2).^24,25,26,27,28,32,33,34,37,38,39,40,41,42,43,44,45,46,47,50,57,60,62,64,66,68^ Furthermore, 9 AIPAs (21%) mentioned the use of AI in their product descriptions but did not offer any specific details about the AI technique applied to develop the model.^37,38,39,42,45,47,48,50,54^ These 9 predictive ML algorithms were FDA approved or CE marked. Twelve of 43 (28%) predictive ML algorithms were implemented (life cycle phase 6) in a research setting but not in practice.^26,31,32,33,34,58,61,62,63,64,66,67^

**Table 2.** Key Characteristics of the 43 Predictive Machine Learning Algorithms in Primary Care Included From Scientific Literature and US Food and Drug Administration and Conformité Européene Registration Databases

Name of predictive ML algorithm	Source	Country of origin	Types of applications	Health condition	AI model	Study design
**From scientific literature**						
Aifred	Benrimoh et al,^58^ 2021	Canada	Treatment decision	Major depression	DL	Usability or acceptability testing
VisualDx	Breitbart et al,^61^ 2020	Germany	Diagnostic decision	Skin lesions	ML	Randomized feasibility study
DSS framework	Frontoni et al,^66^ 2020	Italy	Diagnostic decision support	Type 2 diabetes	ML	Development of a novel framework
ECG-enabled stethoscope	Bachtiger et al,^26^ 2022	UK	Diagnostic decision support	LVEF	DL	Observational, prospective, multicenter study
AI systems to identify diabetic retinopathy	Kanagasingam et al,^62^ 2018	Australia	Referral support	Diabetic retinopathy	ML	Case-control
Automated diabetic retinopathy screening system	Liu et al,^24^ 2021	Canada	Diagnostic decision support	Diabetic retinopathy	DL	Prospective cohort study
Medication reconciliation, iPad-based software tools	Long et al,^63^ 2016	US	Education	Adverse drug events	NLP	Development and observational study
AI for risk prediction glycemic control	Romero-Brufau et al,^64^ 2020	US	Treatment decision support	Diabetes	ML	Usability testing or surveys
A-GPS	Seol et al,^65^ 2021	US	Treatment decision support	Asthma	NLP and ML	A single-center pragmatic RCT with stratified randomization
PULsE-AI	Hill et al,^28^ 2020	UK	Diagnostic decision support	AF	ML	Prospective RCT
ML-based decision support for UTIs	Herter et al,^29^ 2022	The Netherlands	Treatment decision support	UTIs	ML	A routine practice-based prospective observational study design
Risk stratification AI tool	Bhatt et al,^30^ 2021	US	Risk stratification	Population health	ML	Preimplementation and postdeployment evaluation and monitoring
CUHAS-ROBUST	Herman et al,^31^ 2021	Indonesia	Diagnostic decision support	TB screening	DL	A qualitative approach with content analysis
Risk-stratification AI tool AF	Wang et al,^32^ 2019	US	Treatment decision support	AF	ML	Stepped wedge RCT
Personalized BP and lifestyle identification	Chiang et al,^33^2021	US	Treatment decision support	BP	ML	Prospective cohort study
EAGLE	Yao et al,^34^ 2021	US	Diagnostic decision support	Low ejection fraction	ML	Pragmatic RCT
MEDO-Hip	Jaremko et al,^35^ 2023	Canada	Diagnostic decision support	Hip dysplasia	ML	Implementation study
Firstderm	Escalé-Besa et al,^67^ 2023	Spain	Diagnostic decision support	Skin lesions	ML	Prospective, multicenter, observational feasibility study
**From CE and FDA registration databases **						
Tyto Scope	TytoCare,^37^ 2016	US	Diagnostic decision support	Cardiovascular	Unknown	NA
Peerbridge Health	Peerbridge Health,^38^ 2017	US	Diagnostic decision support	Cardiovascular	Unknown	NA
RootiRx	Rooti Labs Limited,^39^ 2016	Taiwan	Diagnostic decision support	Cardiovascular	Unknown	NA
LumineticsCore (formerly IDx-DR)	Digital Diagnostics,^40^ 2018	US	Diagnostic decision support	Diabetic retinopathy	DL	NA
FibriCheck	FibriCheck,^41^ 2018	Belgium	Diagnostic decision support	Cardiovascular	DL	NA
Cardio-HART	Cardio-HART,^42^ 2018	US	Diagnostic decision support	Cardiovascular	Unknown	NA
eMurmur	eMurmur,^43^ 2019	Austria	Diagnostic decision support	Cardiovascular	ML	NA
Smartho-D2 electronic stethoscope	Minttihealth,^44^ 2019	China	Diagnostic decision support	Cardiovascular	DL	NA
Biosticker system	BioIntelliSense,^45^ 2019	US	Monitoring	Cardiovascular	Unknown	NA
EKO analysis software	EKO,^27^ 2020	US	Diagnostic decision support	Cardiovascular	DL	NA
EchoNous (formerly KOSMOS)	EchoNous Inc,^46^ 2020	US	Diagnostic decision support	Cardiovascular	DL	NA
EyeArt	EyeNuk Inc,^25^ 2015	US	Diagnostic decision support	Diabetic retinopathy	DL	NA
Coala heart monitor	Coala,^47^ 2016	Sweden	Diagnostic decision support	Cardiovascular	Unknown	NA
MyAsthma	my mhealth,^48^ 2017	England	Education	Respiratory	Unknown	NA
eMed (formerly Babylon)	eMed,^49^ 2017	England	Diagnostic decision support	General hospital	ML	NA
MedoPad	MedoPad,^50^ 2018	England	Monitoring	Cardiovascular	Unknown	NA
DERM	Skin Analytics,^51^ 2018	England	Diagnostic decision support	Dermatology	DL	NA
ResAppDx-EU	ResApp Health,^52^ 2019	Australia	Diagnostic decision support	Respiratory	ML	NA
AVE (formerly AVEC)	MobileODT,^53^ 2019	Israel	Diagnostic decision support	Obstetrics/Gynecology	ML	NA
Kata	Kata,^54^ 2019	Germany	Education	Respiratory	Unknown	NA
SkinVision	SkinVision,^55^ 2020	The Netherlands	Diagnostic decision support	Dermatology	ML	NA
DeepRhythmAI	Medicalgorithmics,^56^ 2022	Poland	Diagnostic decision support	Cardiovascular	DL	NA
IRNF app	Apple,^57^ 2021	US	Diagnostic decision support	Cardiovascular	ML	NA
Minuteful Kidney	Healthy.io,^59^ 2022	Israel	Diagnostic decision support	Chemistry	ML	NA
Zeus	iRhythm Technologies,^60^ 2022	US	Diagnostic decision support	Cardiovascular	DL	NA

Abbreviations: AF, atrial fibrillation; AI, artificial intelligence; BP, blood pressure; CE, Conformité Européene; DL, deep learning; DSS, decision support system; ECG, electrocardiogram; FDA, US Food and Drug Administration; LVEF, left ventricular ejection fraction; ML, machine learning; NA, not applicable; NLP, natural language processing; RCT, randomized clinical trial; TB, tuberculosis; UTI, urinary tract infection.

### Availability of Evidence of the Predictive ML Algorithms

The search for public availability of evidence on the 43 predictive ML algorithms resulted in 1541 hits, of which 33 were duplicates. Eighty-two publications met the inclusion criteria. Additionally, 80 publications were provided by the product owners or authors or obtained from vendors’ websites. A total of 162 publications were included in the study, which included peer-reviewed articles, technical reports, posters, conference papers, and DMPs (eFigure in Supplement 1). Nine authors and product owners responded and completed the online questionnaire about the reported evidence of the predictive ML algorithm. An overview of the publication characteristics per predictive ML algorithm is provided in eTable 2 in Supplement 1. An overview of the availability of evidence per predictive ML algorithm is provided in eTable 3 in Supplement 1.

### Availability of Evidence per Requirement

**Figure 3.**
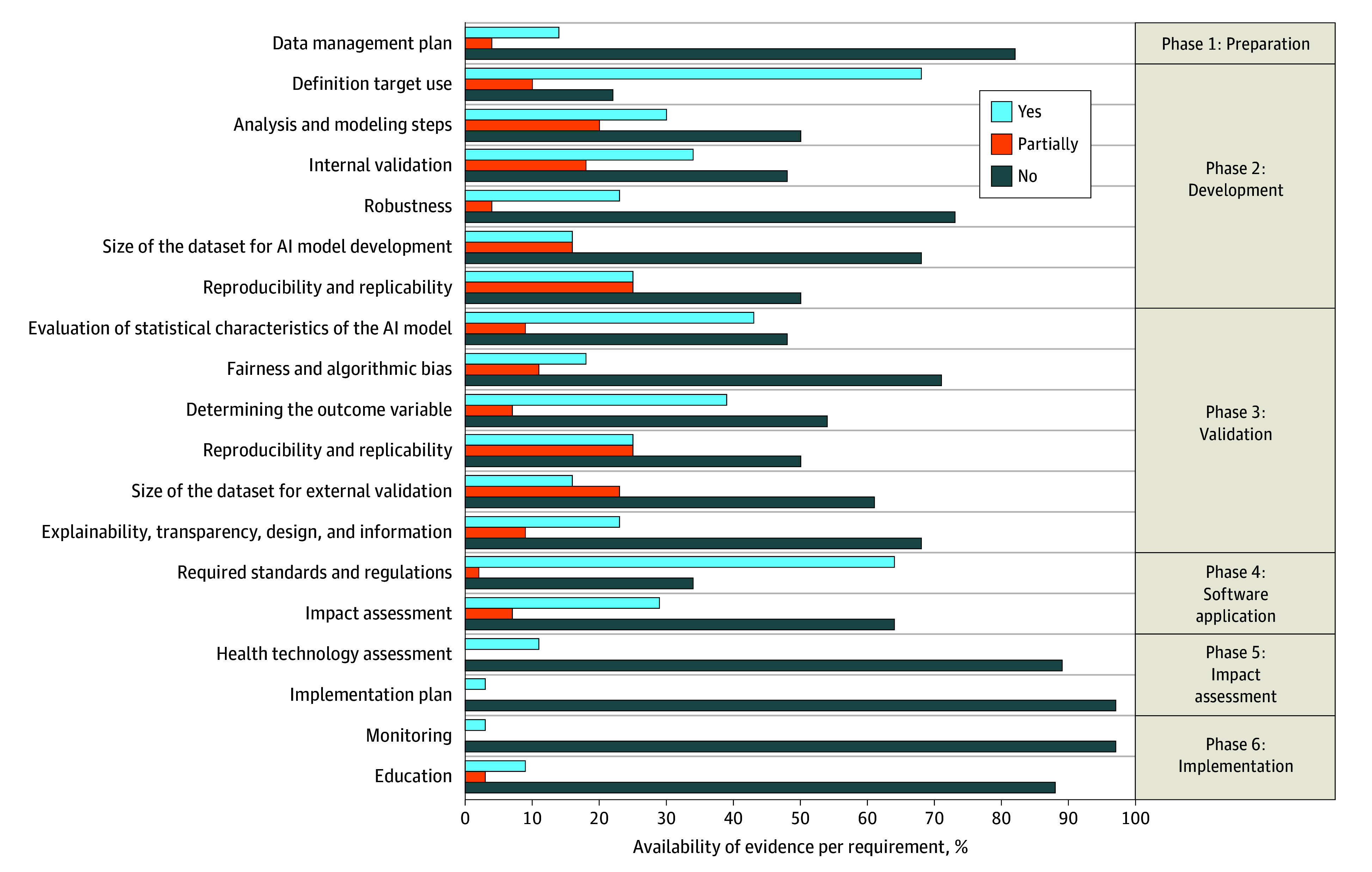
Percentage of Peer-Reviewed Requirements per Phase Light blue indicates the public availability of evidence. Orange indicates that the available evidence partially covered the requirement. Dark blue indicates no evidence was publicly available. AI indicates artificial intelligence.

An overview of the availability of evidence per requirement and according to the life cycle phase is shown in Figure 3. The 3 most commonly available types of evidence per requirement were a clear definition of the target use of the AI model, evaluation of its statistical characteristics, and adherence to software standards and regulations (78%, 47%, and 66% availability, respectively). Conversely, the least available evidence pertained to the implementation plan, monitoring, and health technology assessment (2%, 2%, and 14% availability, respectively), largely due to a lack of information from FDA-approved and CE-marked predictive ML algorithms.

The life cycle phase with the most comprehensive evidence was phase 2 (development), where 46% evidence availability for the relevant requirements was identified. This finding was followed by life cycle phase 3 (validation), with a 39% evidence availability. The life cycle phases with the most limited availability of evidence were phase 1 (preparation) at 19% and phase 5 (impact assessment) at 30%. Commercially available CE-marked and FDA-approved AIPAs offered less evidence across all life cycle phases compared with AIPAs found in the literature database search (29% vs 48% of the overall score to be determined).

### Availability of Evidence per Predictive ML Algorithm

For 5 predictive ML algorithms (12%), evidence was available only for 2 requirements: definition of the target use and required standards and regulations (availability per predictive ML algorithm score: 4 of 48 possible points).^37,38,47,48,69^ Twelve (28%) predictive ML algorithms obtained approximately half of their individual maximum attainable evidence availability score.^24,26,27,28,30,31,33,34,36,41,43,65^ Twelve (28%) did not reach life cycle phase 6 (implementation).^26,31,32,33,34,36,58,61,62,63,64,66^ The predictive ML algorithms that reported the highest availability of evidence per predictive ML algorithm score were a risk-prediction algorithm for identifying undiagnosed atrial fibrillation^28^ (36 of 48 possible points) and an AI-powered clinical decision support tool that enabled early diagnosis of low ejection fraction^34^ (37 of 42 possible points). Both predictive ML algorithms were neither CE-marked nor FDA-approved at the time of publication. Overall, predictive ML algorithms identified through the peer-reviewed literature database search yielded more publicly available evidence^24,26,28,29,30,31,32,33,34,35,36,58,61,62,63,64,65,66^ compared with the predictive ML algorithms identified solely from FDA-approved or CE-marked databases^25,27,37,38,39,40,41,42,43,44,45,46,47,48,49,50,51,52,53,54,55,57,59,60,68^ (45% vs 29%) (eTable 2 in Supplement 1).

## Discussion

To our knowledge, this systematic review provides the most comprehensive overview of predictive ML algorithms implemented in primary care to date and reveals insufficient public availability of evidence of a broad set of predictive ML algorithm quality criteria. The availability of evidence was highly inconsistent across the included predictive ML algorithms, life cycles, and individual quality criteria. Predictive ML algorithms identified from peer-reviewed literature generally provided more publicly available evidence compared with predictive ML algorithms identified solely from FDA or CE registration databases.

The results align with those of previously published research. The scarcity of evidence is particularly pronounced among predictive ML algorithms that have received FDA approval or CE marking.^9,12,13,70,71,72^ Many AI developers in the health care sector are known not to disclose information in the literature about the development, validation, evaluation, or implementation of AI tools.^12,19,73^ There may be tension between protecting intellectual property and being transparent.^74^ Moreover, not all evidence requires peer review, including regulatory processes such as obtaining a CE mark, where notified bodies assess the high-risk medical devices’ evidence for compliance. However, concerns may arise regarding the complexity of methodologies when reporting on effectiveness in a clinical setting. In such cases, there might be a preference for a peer-reviewed process to ensure that evaluation does not solely rely on notified bodies.^75^ Although the FDA and the European Union Medical Device Regulation and, more recently, the AI Act, have released new initiatives to enhance transparency, disclosure of evidence was not mandatory at the time of writing this systematic review.^76,77,78,79,80^ It would be interesting to assess the impact of new regulation in the future.

The availability of evidence fosters transparency and trust among end users, allowing other investigators to scrutinize the data and methods used and thus ensuring ethical and unbiased research and development practices.^81,82^ Researchers can build on previous work, advancing scientific knowledge by making evidence available. If studies lack the necessary details, subsequent researchers may be more likely to create a new AI model instead of validating or updating an existing one. In addition, transparent reporting of predictive ML algorithms encourages vigilance among users, increasing the level of trust humans have in AI, as shown by human factors research.^82^ On the other hand, failing to provide evidence can hamper patient safety due to, for example, algorithmically generated outcomes, interpretations, and recommendations that exhibit unfair advantages or disadvantages for specific individuals or groups.^83^

The results show that evidence was the most scarce regarding the availability of, or reference to, a DMP. The DMP, while not necessarily required to be publicly accessible, is critical to preparing for collecting, managing, and processing data. The DMP plays an overarching role in the entire trajectory toward structurally implementing and using the AI model in daily practice.^84^ It forms an essential component for every stage of the predictive ML algorithm life cycle and can ensure and safeguard data quality, reproducibility, and transparency while striving for Findable, Accessible, Interoperable, and Reusable (FAIR) data.^85,86,87,88^ The FAIR principles aim to support the reuse of scholarly data, including algorithms, and to focus on making data findable and reusable by humans and machines.^87^ Although FAIR principles have been widely adopted in academic contexts, the response of the industry has been less consistent.^89^

Evidence was also limited regarding the impact and health technology assessments of predictive ML algorithms. The lack of accessible evaluations of the outcome and implementation in everyday clinical practice may hinder the translation of research findings into practical applications in health care.^90^ Lack of such information may also impede adoption, as medical professionals need robust evidence to gain trust in these technologies and consistently integrate them into their everyday workflow. Medical professionals stress the importance of adhering to legal, ethical, and quality standards. They voice the need to be trained to interpret the availability of evidence supporting the safety of AI systems, including predictive ML algorithms and their effectiveness.^8,81^ Without this information, it is challenging to ascertain whether the success of a predictive ML algorithm model is attributable to the model itself, the elements of its implementation, or both. As a result, it can be challenging to inform stakeholders about which, how, and for whom predictive ML algorithms are most effective.

### Implications for Research and Practice

Applying the Dutch AIPA guideline requirements to structure the availability of evidence, as demonstrated in this systematic review, can serve as a blueprint for showcasing to policymakers, primary care practitioners, and patients the reliability, transparency, and advancement of predictive ML algorithms. The guideline also has the potential to accelerate the process of complying with regulations.^16^ Although not legally binding, the guideline can be used by developers and researchers as the basis for self-assessment. Furthermore, in the context of Dutch primary care, wherein general practitioners often operate within smaller organizations, limited resources may impede their ability to evaluate complex AI models effectively.^91^ Therefore, comprehensive tools for assessing the availability of evidence on predictive ML algorithms, such as the Dutch AIPA guideline and the practical evaluation tool we developed, are valuable to primary care professionals and may aid large-scale adoption of predictive ML algorithms in practice. Since primary care worldwide is under substantial pressure, from a health systems perspective, it is essential to remove barriers to implementing innovation such as predictive ML algorithms.

### Limitations

This study has methodological limitations that should be taken into account. First, the scope of the systematic review excluded regression-based predictive models and simple rule-based systems. Although these approaches can be of substantial value in primary care, the focus on predictive ML algorithms enabled us to provide an in-depth overview of the aspects of model complexity and interpretability. Although most accepted definitions of ML do not exclude simple regression, the scope was beneficial for maintaining a manageable overview of more complex models that pose unique challenges for standardized reporting of model development, validation, and implementation. Second, we restricted the systematic review to articles published in English. We believe this restriction does not substantially affect the generalizability of the results since previous research has found no evidence of systematic bias due to English-language limitations.^92^ Third, we could not formally compare the predictive validity across predictive ML algorithms due to the substantial variations in the types of AI models and heterogeneous methods between studies. Additionally, the availability scores presented in this study should be seen as an approximation of the degree of public availability of evidence, in line with the objectives described . Fourth, the Dutch AIPA guideline is a local norm, which is not legally binding. Several international AI guidelines exist that apply to predictive ML algorithms, such as TRIPOD+AI (Transparent Reporting of a Multivariable Prediction Model for Individual Prognosis or Diagnosis) for development and validation, DECIDE-AI (Developmental and Exploratory Clinical Investigations of Decision Support Systems Driven by Artificial Intelligence) for feasibility studies, and SPIRIT-AI (Standard Protocol Items: Recommendations for Interventional Trials–Artificial Intelligence) and CONSORT-AI (Consolidated Standards of Reporting Trials–Artificial Intelligence) for impact assessments.^93,94,95,96,97^ These international guidelines, however, are aimed primarily at researchers. We chose to build on the Dutch AIPA guideline because it provides complete, structured, and pragmatic quality assessment derived from existing guidelines across the entire AI life cycle, and it is specific for predictive algorithms, which are considered to be of great potential in the medical field.^15^ It specifically emphasizes implementation aspects and practical applications in clinical practice and may therefore be useful for primary care professionals.

## Conclusions

In this systematic review, we comprehensively identified the availability of evidence of predictive ML algorithms in primary care, using the Dutch AIPA guideline as a reference. We found a scarcity of evidence across the AI life cycle phases for implemented predictive ML algorithms, particularly from algorithms published in FDA-approved or CE-marked databases. Adopting guidelines such as the Dutch AIPA guideline can improve the availability of evidence regarding the predictive ML algorithms’ quality criteria. It could facilitate transparent and consistent reporting of the quality criteria in literature, potentially fostering trust among end users and facilitating large-scale implementation.

## Notes

Supplement 1. **eAppendix 1.** Search Strategies Used Up to July 7, 2023 **eAppendix 2.** Selection Process **eAppendix 3.** Online Questionnaire for Information From Authors and Commercial Product Owners **eTable 1.** The Evidence Requirements Established per Life Cycle Phase as Described in the Dutch AIPA Guideline **eFigure.** Flowchart of Literature Inclusion for Assessment of the Six Phases **eTable 2.** Overview of Publication Characteristics per Predictive ML Algorithm **eTable 3.** Overview of the Availability of Evidence per Predictive ML Algorithm

Supplement 2.
Data Sharing Statement
