# Supplementary material for: Availability of Evidence for Predictive Machine Learning Algorithms in Primary Care: A Systematic Review
Source: JAMA Netw Open. 2024 Sep 12;7(9):e2432990. doi: 10.1001/jamanetworkopen.2024.32990 (PMC11393722; doi:10.1001/jamanetworkopen.2024.32990)
Supplement: Supplement 1. — eAppendix 1. Search Strategies Used Up to July 7, 2023 eAppendix 2. Selection Process eAppendix 3. Online Questionnaire for Information From Authors and Commercial Product Owners eTable 1. The Evidence Requirements Established per Life Cycle Phase as Described in the Dutch AIPA Guideline eFigure. Flowchart of Literature Inclusion for Assessment of the Six Phases eTable 2. Overview of Publication Characteristics per Predictive ML Algorithm eTable 3. Overview of the Availability of Evidence per Predictive ML Algorithm [file jamanetwopen-e2432990-s001.pdf]

Supplemental Online Content

Rakers MM, van Buchem MM, Kucenko S, et al. Availability of evidence for predictive machine learning algorithms in primary care: a systematic review. *JAMA Netw Open*. 2024;7(9):e2432990. doi:10.1001/jamanetworkopen.2024.32990

eAppendix 1. Search Strategies Used Up to July 7, 2023

eAppendix 2. Selection Process

eAppendix 3. Online Questionnaire for Information From Authors and Commercial Product Owners

eTable 1. The Evidence Requirements Established per Life Cycle Phase as Described in the Dutch AIPA Guideline

eFigure. Flowchart of Literature Inclusion for Assessment of the Six Phases

eTable 2. Overview of Publication Characteristics per Predictive ML Algorithm

eTable 3. Overview of the Availability of Evidence per Predictive ML Algorithm

This supplemental material has been provided by the authors to give readers additional information about their work.

eAppendix 1 – Search strategies used up to July 7, 2023

The searches were conducted on the following dates:

- Initial search: April 8, 2021
- Updated search: May 20, 2022
- Second updated search: July 7, 2023

• P (population): Treated in the primary care setting in any country/the Netherlands.

• I (intervention): AI application in pilot phase or implemented in practice use (MDR application p.e.)

• C (comparison): No intervention, standard care, another computer science intervention or any other comparator.

• O (outcomes): any outcome reported.

Database	Query
AI Terms	((("Artificial Intelligence"[majr] OR "Artificial Intelligence"[ti] OR "Biological Ontologies"[ti] OR "Biological Ontology"[ti] OR "Computational Intelligence"[ti] OR "Computer Heuristics"[ti] OR "Computer Intelligence"[ti] OR "Computer Neural Network"[ti] OR "Computer Neural Networks"[ti] OR "Computer Reasoning"[ti] OR "Computer Vision System"[ti] OR "Computer Vision Systems"[ti] OR "Deep Learning"[ti] OR "Machine Intelligence"[ti] OR "Machine Learning"[ti] OR "Natural Language Processing"[ti] OR "Robotic"[ti] OR "Robotics"[ti] OR "Supervised Machine Learning"[ti] OR "Support Vector Machine"[ti] OR "Support Vector Machines"[ti] OR "Unsupervised Machine Learning"[ti] OR "Neural Network"[ti] OR "Neural Networks"[ti] OR "Neural Network*"[ti] OR "Data Mining"[majr] OR "Data Mining"[ti] OR "text mining"[ti] OR "Multifactor Dimensionality Reduction"[ti] OR "Big Data"[majr] OR "Big Data"[ti] OR "Data Science"[majr] OR "Data Science"[ti] OR "Data Sciences"[ti] OR "Data Analytics"[ti] OR "Data driven"[ti] OR "Datasets as Topic"[majr] OR "Bayesian Network"[ti] OR "Bayes Network"[ti] OR "Bayesian Networks"[ti] OR "Bayes Networks"[ti] OR "Bayesian Network"[ti] OR "Bayes Network"[ti] OR "Bayesian Networks"[ti] OR "Bayes Networks"[ti] OR "Bayes Theorem"[majr] OR "Bayes"[ti] OR "Bayesian"[ti]) AND ("Primary Health Care"[Mesh] OR "Primary Care Nursing"[Mesh] OR "Physicians, Primary Care"[Mesh] OR "Physicians, Family"[Mesh] OR "General Practitioners"[Mesh] OR "Family Practice"[Mesh] OR "General Practice"[Mesh] OR "primary healthcare"[tw] OR "Family Practice"[tw] OR "General Practice"[tw] OR "primary care"[tw] OR "primary health care"[tw] OR "Family Physician"[tw] OR "Family Physicians"[tw] OR "Family Doctor"[tw] OR "Family Doctors"[tw] OR "General Practitioner"[tw] OR "General Practitioners"[tw] OR "General Practice Physician"[tw] OR "General Practice Physicians"[tw] OR "Primary Medical Care"[tw] OR "Family Healthcare"[tw] OR "Family Health Care"[tw] OR "Family Medicine"[tw] OR "Nurse Practitioner"[tw] OR "Nurse Practitioners"[tw] OR "Family Nurse"[tw] OR "Family Nurses"[tw] OR "Family Nursing"[tw] OR "Community Medicine"[Mesh] OR "Community Medicine"[tw] OR "Community Practice"[tw] OR "community physician"[tw] OR "community physicians"[tw] OR "community doctor"[tw] OR "community doctor"[tw] OR "community practitioner"[tw] OR "community practitioner"[tw] OR "community nurse"[tw] OR "community nurses"[tw] OR "community nursing"[tw]) AND ("Program Evaluation"[Mesh] OR "Evaluation Study"[ptyp] OR "Evaluation Studies as Topic"[Mesh] OR "Process Assessment, Health Care"[Mesh] OR "Implementation Science"[Mesh] OR "Health Plan Implementation"[Mesh] OR "Pilot Projects"[Mesh] OR "Preliminary Data"[Mesh] OR "Adopt*"[tw] OR "Adoption"[tw] OR "Assess*"[tw] OR "Assessment"[tw] OR "Evaluat*"[tw] OR "Evaluation studies"[tw] OR "Evaluation study"[tw] OR "Evaluation*"[tw] OR "Implement*"[tw] OR "Implementation"[tw] OR "pilot phase"[tw] OR "Pilot Studies"[tw] OR "Pilot Study"[tw] OR "Preliminary Data"[tw] OR

© 2024 Rakers MM et al. *JAMA Network Open*

Database	Query
	"Process Assess*"[tw] OR "Process Assessment"[tw] OR "Process Measure"[tw] OR "Process Measures"[tw] OR "Program Appropriat*"[tw] OR "Program Appropriateness"[tw] OR "Program Assess*"[tw] OR "Program Assessment"[tw] OR "Program Effectiv*"[tw] OR "Program Effectiveness"[tw] OR "Program Evaluat*"[tw] OR "Program Evaluation"[tw] OR "Program Sustain*"[tw] OR "Program Sustainability"[tw] OR "Programme Appropriat*"[tw] OR "Programme Appropriateness"[tw] OR "Programme Assess*"[tw] OR "Programme Assessment"[tw] OR "Programme Effectiv*"[tw] OR "Programme Effectiveness"[tw] OR "Programme Evaluat*"[tw] OR "Programme Evaluation"[tw] OR "Programme Sustain*"[tw] OR "Programme Sustainability"[tw]))
Embase	(((exp *"Artificial Intelligence"/ OR exp *"machine learning"/ OR "Artificial Intelligence".ti OR "Biological Ontologies".ti OR "Biological Ontology".ti OR "Computational Intelligence".ti OR "Computer Heuristics".ti OR "Computer Intelligence".ti OR "Computer Neural Network".ti OR "Computer Neural Networks".ti OR "Computer Reasoning".ti OR "Computer Vision System".ti OR "Computer Vision Systems".ti OR "Deep Learning".ti OR "Machine Intelligence".ti OR "Machine Learning".ti OR "Natural Language Processing".ti OR "Robotic".ti OR "Robotics".ti OR "Supervised Machine Learning".ti OR "Support Vector Machine".ti OR "Support Vector Machines".ti OR "Unsupervised Machine Learning".ti OR "Knowledge Acquisition".ti OR "Knowledge Representation".ti OR "Neural Network".ti OR "Neural Networks".ti OR "Neural Network*".ti OR "Data Mining"/ OR "Data Mining".ti OR "text mining".ti OR "Multifactor Dimensionality Reduction".ti OR "Big Data"/ OR "Big Data".ti OR "Data Science"/ OR "Data Science".ti OR "Data Sciences".ti OR "Data Analytics".ti OR "Data driven".ti OR "Bayesian Network".ti OR "Bayes Network".ti OR "Bayesian Networks".ti OR "Bayes Networks".ti OR "Bayesian Network".ti OR "Bayes Network".ti OR "Bayesian Networks".ti OR "Bayes Networks".ti OR "Bayes Theorem"/) AND (exp *"Primary Health Care"/ OR exp *"Primary Care Nursing"/ OR exp *"general practitioner"/ OR exp *"Physicians, Family"/ OR exp *"General Practitioners"/ OR exp *"Family Practice"/ OR exp *"General Practice"/ OR "primary healthcare".ti,ab OR "Family Practice".ti,ab OR "General Practice".ti,ab OR "primary care".ti,ab OR "primary health care".ti,ab OR "Family Physician".ti,ab OR "Family Physicians".ti,ab OR "Family Doctor".ti,ab OR "Family Doctors".ti,ab OR "General Practitioner".ti,ab OR "General Practitioners".ti,ab OR "General Practice Physician".ti,ab OR "General Practice Physicians".ti,ab OR "Primary Medical Care".ti,ab OR "Family Healthcare".ti,ab OR "Family Health Care".ti,ab OR "Family Medicine".ti,ab OR "Nurse Practitioner".ti,ab OR "Nurse Practitioners".ti,ab OR "Family Nurse".ti,ab OR "Family Nurses".ti,ab OR "Family Nursing".ti,ab OR exp *"Community Medicine"/ OR "Community Medicine".ti,ab OR "Community Practice".ti,ab OR "community physician".ti,ab OR "community physicians".ti,ab OR "community doctor".ti,ab OR "community doctor".ti,ab OR "community practitioner".ti,ab OR "community practitioner".ti,ab OR "community nurse".ti,ab OR "community nurses".ti,ab OR "community nursing".ti,ab) AND (exp *"Program Evaluation"/ OR exp *"Evaluation Study"/ OR exp *"Evaluation Studies as Topic"/ OR exp *"Process Assessment, Health Care"/ OR exp *"Implementation Science"/ OR exp *"Health Plan Implementation"/ OR exp *"Pilot Projects"/ OR exp *"Pilot Study"/ OR exp *"Preliminary Data"/ OR "Adopt*".ti,ab OR "Adoption".ti,ab OR "Assess*".ti,ab OR "Assessment".ti,ab OR "Evaluat*".ti,ab OR "Evaluation studies".ti,ab OR "Evaluation study".ti,ab OR "Evaluation*".ti,ab OR "Implement*".ti,ab OR "Implementation".ti,ab OR "pilot phase".ti,ab OR "Pilot Studies".ti,ab OR "Pilot Study".ti,ab OR "Preliminary Data".ti,ab OR "Process Assess*".ti,ab OR "Process Assessment".ti,ab OR "Process Measure".ti,ab OR "Process Measures".ti,ab OR "Program Appropriat*".ti,ab OR "Program Appropriateness".ti,ab OR "Program Assess*".ti,ab OR "Program Assessment".ti,ab OR "Program Effectiv*".ti,ab OR "Program Effectiveness".ti,ab OR "Program Evaluat*".ti,ab OR "Program

Database	Query
	Evaluation".ti,ab OR "Program Sustain*".ti,ab OR "Program Sustainability".ti,ab OR "Programme Appropriat*".ti,ab OR "Programme Appropriateness".ti,ab OR "Programme Assess*".ti,ab OR "Programme Assessment".ti,ab OR "Programme Effectiv*".ti,ab OR "Programme Effectiveness".ti,ab OR "Programme Evaluat*".ti,ab OR "Programme Evaluation".ti,ab OR "Programme Sustain*".ti,ab OR "Programme Sustainability".ti,ab))
Web of Science	((ti=("Artificial Intelligence" OR "machine learning" OR "Artificial Intelligence" OR "Biological Ontologies" OR "Biological Ontology" OR "Computational Intelligence" OR "Computer Heuristics" OR "Computer Intelligence" OR "Computer Neural Network" OR "Computer Neural Networks" OR "Computer Reasoning" OR "Computer Vision System" OR "Computer Vision Systems" OR "Deep Learning" OR "Machine Intelligence" OR "Machine Learning" OR "Natural Language Processing" OR "Robotic" OR "Robotics" OR "Supervised Machine Learning" OR "Support Vector Machine" OR "Support Vector Machines" OR "Unsupervised Machine Learning" OR "Knowledge Acquisition" OR "Knowledge Representation" OR "Neural Network" OR "Neural Networks" OR "Neural Network*" OR "Data Mining" OR "Data Mining" OR "text mining" OR "Multifactor Dimensionality Reduction" OR "Big Data" OR "Big Data" OR "Data Science" OR "Data Science" OR "Data Sciences" OR "Data Analytics" OR "Data driven" OR "Datasets as Topic" OR "Bayesian Network" OR "Bayes Network" OR "Bayesian Networks" OR "Bayes Networks" OR "Bayesian Network" OR "Bayes Network" OR "Bayesian Networks" OR "Bayes Networks" OR "Bayes Theorem") AND ts=("Primary Health Care" OR "Primary Care Nursing" OR "general practitioner" OR "Physicians, Family" OR "General Practitioners" OR "Family Practice" OR "General Practice" OR "primary healthcare" OR "Family Practice" OR "General Practice" OR "primary care" OR "primary health care" OR "Family Physician" OR "Family Physicians" OR "Family Doctor" OR "Family Doctors" OR "General Practitioner" OR "General Practitioners" OR "General Practice Physician" OR "General Practice Physicians" OR "Primary Medical Care" OR "Family Healthcare" OR "Family Health Care" OR "Family Medicine" OR "Nurse Practitioner" OR "Nurse Practitioners" OR "Family Nurse" OR "Family Nurses" OR "Family Nursing" OR "Community Medicine" OR "Community Medicine" OR "Community Practice" OR "community physician" OR "community physicians" OR "community doctor" OR "community doctor" OR "community practitioner" OR "community practitioner" OR "community nurse" OR "community nurses" OR "community nursing") AND ts=("Program Evaluation" OR "Evaluation Study" OR "Evaluation Studies as Topic" OR "Process Assessment, Health Care" OR "Implementation Science" OR "Health Plan Implementation" OR "Pilot Projects" OR "Pilot Study" OR "Preliminary Data" OR "Adopt*" OR "Adoption" OR "Assess*" OR "Assessment" OR "Evaluat*" OR "Evaluation studies" OR "Evaluation study" OR "Evaluation*" OR "Implement*" OR "Implementation" OR "pilot phase" OR "Pilot Studies" OR "Pilot Study" OR "Preliminary Data" OR "Process Assess*" OR "Process Assessment" OR "Process Measure" OR "Process Measures" OR "Program Appropriat*" OR "Program Appropriateness" OR "Program Assess*" OR "Program Assessment" OR "Program Effectiv*" OR "Program Effectiveness" OR "Program Evaluat*" OR "Program Evaluation" OR "Program Sustain*" OR "Program Sustainability" OR "Programme Appropriat*" OR "Programme Appropriateness" OR "Programme Assess*" OR "Programme Assessment" OR "Programme Effectiv*" OR "Programme Effectiveness" OR "Programme Evaluat*" OR "Programme Evaluation" OR "Programme Sustain*" OR "Programme Sustainability"))
Cochrane	((("Artificial Intelligence" OR "machine learning" OR "Artificial Intelligence" OR "Biological Ontologies" OR "Biological Ontology" OR "Computational Intelligence" OR "Computer Heuristics" OR "Computer Intelligence" OR "Computer Neural Network" OR "Computer Neural Networks" OR "Computer Reasoning" OR "Computer Vision System" OR "Computer Vision Systems" OR

© 2024 Rakers MM et al. *JAMA Network Open*

Database	Query
	"Deep Learning" OR "Expert System" OR "Expert Systems" OR "Fuzzy Logic" OR "Hierarchical Learning" OR "Knowledge Base" OR "Knowledge Bases" OR "Machine Intelligence" OR "Machine Learning" OR "Natural Language Processing" OR "Robotic" OR "Robotics" OR "Supervised Machine Learning" OR "Support Vector Machine" OR "Support Vector Machines" OR "Unsupervised Machine Learning" OR "Knowledge Acquisition" OR "Knowledge Representation" OR "Neural Network" OR "Neural Networks" OR "Neural Network*" OR "Data Mining" OR "Data Mining" OR "text mining" OR "Multifactor Dimensionality Reduction" OR "Big Data" OR "Big Data" OR "Data Science" OR "Data Science" OR "Data Sciences" OR "Data Analytics" OR "Data driven" OR "Datasets as Topic" OR "Bayesian Network" OR "Bayes Network" OR "Bayesian Networks" OR "Bayes Networks" OR "Bayesian Network" OR "Bayes Network" OR "Bayesian Networks" OR "Bayes Networks" OR "Bayes Theorem"):ti AND ("Primary Health Care" OR "Primary Care Nursing" OR "general practitioner" OR "Physicians, Family" OR "General Practitioners" OR "Family Practice" OR "General Practice" OR "primary healthcare" OR "Family Practice" OR "General Practice" OR "primary care" OR "primary health care" OR "Family Physician" OR "Family Physicians" OR "Family Doctor" OR "Family Doctors" OR "General Practitioner" OR "General Practitioners" OR "General Practice Physician" OR "General Practice Physicians" OR "Primary Medical Care" OR "Family Healthcare" OR "Family Health Care" OR "Family Medicine" OR "Nurse Practitioner" OR "Nurse Practitioners" OR "Family Nurse" OR "Family Nurses" OR "Family Nursing" OR "Community Medicine" OR "Community Medicine" OR "Community Practice" OR "community physician" OR "community physicians" OR "community doctor" OR "community doctor" OR "community practitioner" OR "community practitioner" OR "community nurse" OR "community nurses" OR "community nursing"):ti,ab,kw AND ("Program Evaluation" OR "Evaluation Study" OR "Evaluation Studies as Topic" OR "Process Assessment, Health Care" OR "Implementation Science" OR "Health Plan Implementation" OR "Pilot Projects" OR "Pilot Study" OR "Preliminary Data" OR "Adopt*" OR "Adoption" OR "Assess*" OR "Assessment" OR "Evaluat*" OR "Evaluation studies" OR "Evaluation study" OR "Evaluation*" OR "Implement*" OR "Implementation" OR "pilot phase" OR "Pilot Studies" OR "Pilot Study" OR "Preliminary Data" OR "Process Assess*" OR "Process Assessment" OR "Process Measure" OR "Process Measures" OR "Program Appropriat*" OR "Program Appropriateness" OR "Program Assess*" OR "Program Assessment" OR "Program Effectiv*" OR "Program Effectiveness" OR "Program Evaluat*" OR "Program Evaluation" OR "Program Sustain*" OR "Program Sustainability" OR "Programme Appropriat*" OR "Programme Appropriateness" OR "Programme Assess*" OR "Programme Assessment" OR "Programme Effectiv*" OR "Programme Effectiveness" OR "Programme Evaluat*" OR "Programme Evaluation" OR "Programme Sustain*" OR "Programme Sustainability"):ti,ab,kw)
Emcare	(((exp *"Artificial Intelligence"/ OR exp *"machine learning"/ OR "Artificial Intelligence".ti OR "Biological Ontologies".ti OR "Biological Ontology".ti OR "Computational Intelligence".ti OR "Computer Heuristics".ti OR "Computer Intelligence".ti OR "Computer Neural Network".ti OR "Computer Neural Networks".ti OR "Computer Reasoning".ti OR "Computer Vision System".ti OR "Computer Vision Systems".ti OR "Deep Learning".ti OR "Expert System".ti OR "Expert Systems".ti OR "Fuzzy Logic".ti OR "Hierarchical Learning".ti OR "Knowledge Base".ti OR "Knowledge Bases".ti OR "Machine Intelligence".ti OR "Machine Learning".ti OR "Natural Language Processing".ti OR "Robotic".ti OR "Robotics".ti OR "Supervised Machine Learning".ti OR "Support Vector Machine".ti OR "Support Vector Machines".ti OR "Unsupervised Machine Learning".ti OR "Knowledge Acquisition".ti OR "Knowledge Representation".ti OR "Neural Network".ti OR "Neural Networks".ti OR "Neural Network*".ti OR "Data Mining"/ OR "Data Mining".ti OR "text mining".ti OR "Multifactor Dimensionality Reduction".ti OR "Big

© 2024 Rakers MM et al. *JAMA Network Open*

Database	Query
	Data"/ OR "Big Data".ti OR "Data Science"/ OR "Data Science".ti OR "Data Sciences".ti OR "Data Analytics".ti OR "Data driven".ti OR "Bayesian Network".ti OR "Bayes Network".ti OR "Bayesian Networks".ti OR "Bayes Networks".ti OR "Bayesian Network".ti OR "Bayes Network".ti OR "Bayesian Networks".ti OR "Bayes Networks".ti OR "Bayes Theorem"/) AND (exp *"Primary Health Care"/ OR exp *"Primary Care Nursing"/ OR exp *"general practitioner"/ OR exp *"Physicians, Family"/ OR exp *"General Practitioners"/ OR exp *"Family Practice"/ OR exp *"General Practice"/ OR "primary healthcare".ti,ab OR "Family Practice".ti,ab OR "General Practice".ti,ab OR "primary care".ti,ab OR "primary health care".ti,ab OR "Family Physician".ti,ab OR "Family Physicians".ti,ab OR "Family Doctor".ti,ab OR "Family Doctors".ti,ab OR "General Practitioner".ti,ab OR "General Practitioners".ti,ab OR "General Practice Physician".ti,ab OR "General Practice Physicians".ti,ab OR "Primary Medical Care".ti,ab OR "Family Healthcare".ti,ab OR "Family Health Care".ti,ab OR "Family Medicine".ti,ab OR "Nurse Practitioner".ti,ab OR "Nurse Practitioners".ti,ab OR "Family Nurse".ti,ab OR "Family Nurses".ti,ab OR "Family Nursing".ti,ab OR exp *"Community Medicine"/ OR "Community Medicine".ti,ab OR "Community Practice".ti,ab OR "community physician".ti,ab OR "community physicians".ti,ab OR "community doctor".ti,ab OR "community doctor".ti,ab OR "community practitioner".ti,ab OR "community practitioner".ti,ab OR "community nurse".ti,ab OR "community nurses".ti,ab OR "community nursing".ti,ab) AND (exp *"Program Evaluation"/ OR exp *"Evaluation Study"/ OR exp *"Evaluation Studies as Topic"/ OR exp *"Process Assessment, Health Care"/ OR exp *"Implementation Science"/ OR exp *"Health Plan Implementation"/ OR exp *"Pilot Projects"/ OR exp *"Pilot Study"/ OR exp *"Preliminary Data"/ OR "Adopt*".ti,ab OR "Adoption".ti,ab OR "Assess*".ti,ab OR "Assessment".ti,ab OR "Evaluat*".ti,ab OR "Evaluation studies".ti,ab OR "Evaluation study".ti,ab OR "Evaluation*".ti,ab OR "Implement*".ti,ab OR "Implementation".ti,ab OR "pilot phase".ti,ab OR "Pilot Studies".ti,ab OR "Pilot Study".ti,ab OR "Preliminary Data".ti,ab OR "Process Assess*".ti,ab OR "Process Assessment".ti,ab OR "Process Measure".ti,ab OR "Process Measures".ti,ab OR "Program Appropriat*".ti,ab OR "Program Appropriateness".ti,ab OR "Program Assess*".ti,ab OR "Program Assessment".ti,ab OR "Program Effectiv*".ti,ab OR "Program Effectiveness".ti,ab OR "Program Evaluat*".ti,ab OR "Program Evaluation".ti,ab OR "Program Sustain*".ti,ab OR "Program Sustainability".ti,ab OR "Programme Appropriat*".ti,ab OR "Programme Appropriateness".ti,ab OR "Programme Assess*".ti,ab OR "Programme Assessment".ti,ab OR "Programme Effectiv*".ti,ab OR "Programme Effectiveness".ti,ab OR "Programme Evaluat*".ti,ab OR "Programme Evaluation".ti,ab OR "Programme Sustain*".ti,ab OR "Programme Sustainability".ti,ab)) OR ((exp *"Artificial Intelligence"/ OR exp *"machine learning"/ OR "Artificial Intelligence".ti,ab OR "Biological Ontologies".ti,ab OR "Biological Ontology".ti,ab OR "Computational Intelligence".ti,ab OR "Computer Heuristics".ti,ab OR "Computer Intelligence".ti,ab OR "Computer Neural Network".ti,ab OR "Computer Neural Networks".ti,ab OR "Computer Reasoning".ti,ab OR "Computer Vision System".ti,ab OR "Computer Vision Systems".ti,ab OR "Deep Learning".ti,ab OR "Expert System".ti,ab OR "Expert Systems".ti,ab OR "Fuzzy Logic".ti,ab OR "Hierarchical Learning".ti,ab OR "Knowledge Base".ti,ab OR "Knowledge Bases".ti,ab OR "Machine Intelligence".ti,ab OR "Machine Learning".ti,ab OR "Natural Language Processing".ti,ab OR "Robotic".ti,ab OR "Robotics".ti,ab OR "Supervised Machine Learning".ti,ab OR "Support Vector Machine".ti,ab OR "Support Vector Machines".ti,ab OR "Unsupervised Machine Learning".ti,ab OR "Knowledge Acquisition".ti,ab OR "Knowledge Representation".ti,ab OR "Neural Network".ti,ab OR "Neural Networks".ti,ab OR "Neural Network*".ti,ab OR "Data Mining"/ OR "Data Mining".ti,ab OR "text mining".ti,ab OR "Multifactor Dimensionality Reduction".ti,ab OR "Big Data"/ OR "Big Data".ti,ab OR "Data Science"/ OR "Data Science".ti,ab OR "Data

Database	Query
	Sciences".ti,ab OR "Data Analytics".ti,ab OR "Data driven".ti,ab OR "Bayesian Network".ti,ab OR "Bayes Network".ti,ab OR "Bayesian Networks".ti,ab OR "Bayes Networks".ti,ab OR "Bayesian Network".ti,ab OR "Bayes Network".ti,ab OR "Bayesian Networks".ti,ab OR "Bayes Networks".ti,ab OR "Bayes Theorem"/) AND (exp *"Primary Health Care"/ OR exp *"Primary Care Nursing"/ OR exp *"general practitioner"/ OR exp *"Physicians, Family"/ OR exp *"General Practitioners"/ OR exp *"Family Practice"/ OR exp *"General Practice"/ OR "primary healthcare".ti OR "Family Practice".ti OR "General Practice".ti OR "primary care".ti OR "primary health care".ti OR "Family Physician".ti OR "Family Physicians".ti OR "Family Doctor".ti OR "Family Doctors".ti OR "General Practitioner".ti OR "General Practitioners".ti OR "General Practice Physician".ti OR "General Practice Physicians".ti OR "Primary Medical Care".ti OR "Family Healthcare".ti OR "Family Health Care".ti OR "Family Medicine".ti OR "Nurse Practitioner".ti OR "Nurse Practitioners".ti OR "Family Nurse".ti OR "Family Nurses".ti OR "Family Nursing".ti OR exp *"Community Medicine"/ OR "Community Medicine".ti OR "Community Practice".ti OR "community physician".ti OR "community physicians".ti OR "community doctor".ti OR "community doctor".ti OR "community practitioner".ti OR "community practitioner".ti OR "community nurse".ti OR "community nurses".ti OR "community nursing".ti) AND (exp *"Program Evaluation"/ OR exp *"Evaluation Study"/ OR exp *"Evaluation Studies as Topic"/ OR exp *"Process Assessment, Health Care"/ OR exp *"Implementation Science"/ OR exp *"Health Plan Implementation"/ OR exp *"Pilot Projects"/ OR exp *"Pilot Study"/ OR exp *"Preliminary Data"/ OR "Adopt*".ti,ab OR "Adoption".ti,ab OR "Assess*".ti,ab OR "Assessment".ti,ab OR "Evaluat*".ti,ab OR "Evaluation studies".ti,ab OR "Evaluation study".ti,ab OR "Evaluation*".ti,ab OR "Implement*".ti,ab OR "Implementation".ti,ab OR "pilot phase".ti,ab OR "Pilot Studies".ti,ab OR "Pilot Study".ti,ab OR "Preliminary Data".ti,ab OR "Process Assess*".ti,ab OR "Process Assessment".ti,ab OR "Process Measure".ti,ab OR "Process Measures".ti,ab OR "Program Appropriat*".ti,ab OR "Program Appropriateness".ti,ab OR "Program Assess*".ti,ab OR "Program Assessment".ti,ab OR "Program Effectiv*".ti,ab OR "Program Effectiveness".ti,ab OR "Program Evaluat*".ti,ab OR "Program Evaluation".ti,ab OR "Program Sustain*".ti,ab OR "Program Sustainability".ti,ab OR "Programme Appropriat*".ti,ab OR "Programme Appropriateness".ti,ab OR "Programme Assess*".ti,ab OR "Programme Assessment".ti,ab OR "Programme Effectiv*".ti,ab OR "Programme Effectiveness".ti,ab OR "Programme Evaluat*".ti,ab OR "Programme Evaluation".ti,ab OR "Programme Sustain*".ti,ab OR "Programme Sustainability".ti,ab)) OR ((exp *"Artificial Intelligence"/ OR exp *"machine learning"/ OR "Artificial Intelligence".ti OR "Biological Ontologies".ti OR "Biological Ontology".ti OR "Computational Intelligence".ti OR "Computer Heuristics".ti OR "Computer Intelligence".ti OR "Computer Neural Network".ti OR "Computer Neural Networks".ti OR "Computer Reasoning".ti OR "Computer Vision System".ti OR "Computer Vision Systems".ti OR "Deep Learning".ti OR "Expert System".ti OR "Expert Systems".ti OR "Fuzzy Logic".ti OR "Hierarchical Learning".ti OR "Knowledge Base".ti OR "Knowledge Bases".ti OR "Machine Intelligence".ti OR "Machine Learning".ti OR "Natural Language Processing".ti OR "Robotic".ti OR "Robotics".ti OR "Supervised Machine Learning".ti OR "Support Vector Machine".ti OR "Support Vector Machines".ti OR "Unsupervised Machine Learning".ti OR "Knowledge Acquisition".ti OR "Knowledge Representation".ti OR "Neural Network".ti OR "Neural Networks".ti OR "Neural Network*".ti OR "Data Mining"/ OR "Data Mining".ti OR "text mining".ti OR "Multifactor Dimensionality Reduction".ti OR "Big Data"/ OR "Big Data".ti OR "Data Science"/ OR "Data Science".ti OR "Data Sciences".ti OR "Data Analytics".ti OR "Data driven".ti OR "Bayesian Network".ti OR "Bayes Network".ti OR "Bayesian Networks".ti OR "Bayes Networks".ti OR "Bayesian Network".ti OR "Bayes Network".ti OR "Bayesian Networks".ti OR "Bayes Networks".ti OR

© 2024 Rakers MM et al. *JAMA Network Open*

Database	Query
	"Bayes Theorem"/) AND (exp *"Primary Health Care"/ OR exp *"Primary Care Nursing"/ OR exp *"general practitioner"/ OR exp *"Physicians, Family"/ OR exp *"General Practitioners"/ OR exp *"Family Practice"/ OR exp *"General Practice"/ OR "primary healthcare".ti OR "Family Practice".ti OR "General Practice".ti OR "primary care".ti OR "primary health care".ti OR "Family Physician".ti OR "Family Physicians".ti OR "Family Doctor".ti OR "Family Doctors".ti OR "General Practitioner".ti OR "General Practitioners".ti OR "General Practice Physician".ti OR "General Practice Physicians".ti OR "Primary Medical Care".ti OR "Family Healthcare".ti OR "Family Health Care".ti OR "Family Medicine".ti OR "Nurse Practitioner".ti OR "Nurse Practitioners".ti OR "Family Nurse".ti OR "Family Nurses".ti OR "Family Nursing".ti OR exp *"Community Medicine"/ OR "Community Medicine".ti OR "Community Practice".ti OR "community physician".ti OR "community physicians".ti OR "community doctor".ti OR "community doctor".ti OR "community practitioner".ti OR "community practitioner".ti OR "community nurse".ti OR "community nurses".ti OR "community nursing".ti)))
Academic Search Premier	((TI("Artificial Intelligence" OR "machine learning" OR "Artificial Intelligence" OR "Biological Ontologies" OR "Biological Ontology" OR "Computational Intelligence" OR "Computer Heuristics" OR "Computer Intelligence" OR "Computer Neural Network" OR "Computer Neural Networks" OR "Computer Reasoning" OR "Computer Vision System" OR "Computer Vision Systems" OR "Deep Learning" OR "Expert System" OR "Expert Systems" OR "Fuzzy Logic" OR "Hierarchical Learning" OR "Knowledge Base" OR "Knowledge Bases" OR "Machine Intelligence" OR "Machine Learning" OR "Natural Language Processing" OR "Robotic" OR "Robotics" OR "Supervised Machine Learning" OR "Support Vector Machine" OR "Support Vector Machines" OR "Unsupervised Machine Learning" OR "Knowledge Acquisition" OR "Knowledge Representation" OR "Neural Network" OR "Neural Networks" OR "Neural Network*" OR "Data Mining" OR "Data Mining" OR "text mining" OR "Multifactor Dimensionality Reduction" OR "Big Data" OR "Big Data" OR "Data Science" OR "Data Science" OR "Data Sciences" OR "Data Analytics" OR "Data driven" OR "Datasets as Topic" OR "Bayesian Network" OR "Bayes Network" OR "Bayesian Networks" OR "Bayes Networks" OR "Bayesian Network" OR "Bayes Network" OR "Bayesian Networks" OR "Bayes Networks" OR "Bayes Theorem") AND KW("Primary Health Care" OR "Primary Care Nursing" OR "general practitioner" OR "Physicians, Family" OR "General Practitioners" OR "Family Practice" OR "General Practice" OR "primary healthcare" OR "Family Practice" OR "General Practice" OR "primary care" OR "primary health care" OR "Family Physician" OR "Family Physicians" OR "Family Doctor" OR "Family Doctors" OR "General Practitioner" OR "General Practitioners" OR "General Practice Physician" OR "General Practice Physicians" OR "Primary Medical Care" OR "Family Healthcare" OR "Family Health Care" OR "Family Medicine" OR "Nurse Practitioner" OR "Nurse Practitioners" OR "Family Nurse" OR "Family Nurses" OR "Family Nursing" OR "Community Medicine" OR "Community Medicine" OR "Community Practice" OR "community physician" OR "community physicians" OR "community doctor" OR "community doctor" OR "community practitioner" OR "community practitioner" OR "community nurse" OR "community nurses" OR "community nursing") AND KW("Program Evaluation" OR "Evaluation Study" OR "Evaluation Studies as Topic" OR "Process Assessment, Health Care" OR "Implementation Science" OR "Health Plan Implementation" OR "Pilot Projects" OR "Pilot Study" OR "Preliminary Data" OR "Adopt*" OR "Adoption" OR "Assess*" OR "Assessment" OR "Evaluat*" OR "Evaluation studies" OR "Evaluation study" OR "Evaluation*" OR "Implement*" OR "Implementation" OR "pilot phase" OR "Pilot Studies" OR "Pilot Study" OR "Preliminary Data" OR "Process Assess*" OR "Process Assessment" OR "Process Measure" OR "Process Measures" OR "Program Appropriat*" OR "Program Appropriateness" OR "Program Assess*" OR "Program Assessment" OR "Program Effectiv*" OR "Program Effectiveness" OR "Program Evaluat*"

Database	Query
	OR "Program Evaluation" OR "Program Sustain*" OR "Program Sustainability" OR "Programme Appropriat*" OR "Programme Appropriateness" OR "Programme Assess*" OR "Programme Assessment" OR "Programme Effectiv*" OR "Programme Effectiveness" OR "Programme Evaluat*" OR "Programme Evaluation" OR "Programme Sustain*" OR "Programme Sustainability"))
IEEE Xplore	("Artificial Intelligence" OR "machine learning" OR "Deep Learning" OR "Expert System" OR "Data Mining" OR "text mining" OR "Big Data" OR "Data Science" OR "Data Analytics" OR "Data driven" OR "Bayesian" OR "Bayes") AND ("Primary Health Care" OR "Primary Care" OR "general practitioner" OR "Family Practice" OR "General Practice" OR "primary healthcare" OR "Community Medicine" OR "Community Practice" OR "community physician" OR "community nursing")
ACM Digital Libary	("Artificial Intelligence" OR "machine learning" OR "Artificial Intelligence" OR "Biological Ontologies" OR "Biological Ontology" OR "Computational Intelligence" OR "Computer Heuristics" OR "Computer Intelligence" OR "Computer Neural Network" OR "Computer Neural Networks" OR "Computer Reasoning" OR "Computer Vision System" OR "Computer Vision Systems" OR "Deep Learning" OR "Expert System" OR "Expert Systems" OR "Fuzzy Logic" OR "Hierarchical Learning" OR "Knowledge Base" OR "Knowledge Bases" OR "Machine Intelligence" OR "Machine Learning" OR "Natural Language Processing" OR "Robotic" OR "Robotics" OR "Supervised Machine Learning" OR "Support Vector Machine" OR "Support Vector Machines" OR "Unsupervised Machine Learning" OR "Knowledge Acquisition" OR "Knowledge Representation" OR "Neural Network" OR "Neural Networks" OR "Neural Network" OR "Data Mining" OR "Data Mining" OR "text mining" OR "Multifactor Dimensionality Reduction" OR "Big Data" OR "Big Data" OR "Data Science" OR "Data Science" OR "Data Sciences" OR "Data Analytics" OR "Data driven" OR "Bayesian Network" OR "Bayes Network" OR "Bayesian Networks" OR "Bayes Networks" OR "Bayesian Network" OR "Bayes Network" OR "Bayesian Networks" OR "Bayes Networks" OR "Bayes Theorem")
MathSciNet	("Artificial Intelligence" OR "machine learning" OR "Artificial Intelligence" OR "Biological Ontologies" OR "Biological Ontology" OR "Computational Intelligence" OR "Computer Heuristics" OR "Computer Intelligence" OR "Computer Neural Network" OR "Computer Neural Networks" OR "Computer Reasoning" OR "Computer Vision System" OR "Computer Vision Systems" OR "Deep Learning" OR "Expert System" OR "Expert Systems" OR "Fuzzy Logic" OR "Hierarchical Learning" OR "Knowledge Base" OR "Knowledge Bases" OR "Machine Intelligence" OR "Machine Learning" OR "Natural Language Processing" OR "Robotic" OR "Robotics" OR "Supervised Machine Learning" OR "Support Vector Machine" OR "Support Vector Machines" OR "Unsupervised Machine Learning" OR "Knowledge Acquisition" OR "Knowledge Representation" OR "Neural Network" OR "Neural Networks" OR "Neural Network" OR "Data Mining" OR "Data Mining" OR "text mining" OR "Multifactor Dimensionality Reduction" OR "Big Data" OR "Big Data" OR "Data Science" OR "Data Science" OR "Data Sciences" OR "Data Analytics" OR "Data driven" OR "Bayesian Network" OR "Bayes Network" OR "Bayesian Networks" OR "Bayes Networks" OR "Bayesian Network" OR "Bayes Network" OR "Bayesian Networks" OR "Bayes Networks" OR "Bayes Theorem")
Europe PMC	(TITLE:"Artificial Intelligence" OR TITLE:"machine learning" OR TITLE:"Deep Learning" OR TITLE:"Expert System" OR TITLE:"Data Mining" OR TITLE:"text mining" OR TITLE:"Big Data" OR TITLE:"Data Science" OR TITLE:"Data Analytics" OR TITLE:"Data driven" OR TITLE:"Bayesian" OR TITLE:"Bayes") AND (TITLE:"Primary Health Care" OR TITLE:"Primary Care" OR TITLE:"general practitioner" OR TITLE:"Family Practice" OR TITLE:"General Practice" OR TITLE:"primary healthcare" OR TITLE:"Community Medicine" OR TITLE:"Community Practice" OR

© 2024 Rakers MM et al. *JAMA Network Open*

Database	Query
	TITLE:"community physician" OR TITLE:"community nursing") AND (SRC:PPR)<br><br>(TITLE:"Artificial Intelligence" OR TITLE:"machine learning" OR TITLE:"Deep Learning" OR TITLE:"Expert System" OR TITLE:"Data Mining" OR TITLE:"text mining" OR TITLE:"Big Data" OR TITLE:"Data Science" OR TITLE:"Data Analytics" OR TITLE:"Data driven" OR TITLE:"Bayesian" OR TITLE:"Bayes") AND (ABSTRACT:"Primary Health Care" OR ABSTRACT:"Primary Care" OR ABSTRACT:"general practitioner" OR ABSTRACT:"Family Practice" OR ABSTRACT:"General Practice" OR ABSTRACT:"primary healthcare" OR ABSTRACT:"Community Medicine" OR ABSTRACT:"Community Practice" OR ABSTRACT:"community physician" OR ABSTRACT:"community nursing") AND (SRC:PPR)<br><br>(ABSTRACT:"Artificial Intelligence" OR ABSTRACT:"machine learning" OR ABSTRACT:"Deep Learning" OR ABSTRACT:"Expert System" OR ABSTRACT:"Data Mining" OR ABSTRACT:"text mining" OR ABSTRACT:"Big Data" OR ABSTRACT:"Data Science" OR ABSTRACT:"Data Analytics" OR ABSTRACT:"Data driven" OR ABSTRACT:"Bayesian" OR ABSTRACT:"Bayes") AND (TITLE:"Primary Health Care" OR TITLE:"Primary Care" OR TITLE:"general practitioner" OR TITLE:"Family Practice" OR TITLE:"General Practice" OR TITLE:"primary healthcare" OR TITLE:"Community Medicine" OR TITLE:"Community Practice" OR TITLE:"community physician" OR TITLE:"community nursing") AND (SRC:PPR)<br><br>(ABSTRACT:"Artificial Intelligence" OR ABSTRACT:"machine learning" OR ABSTRACT:"Deep Learning" OR ABSTRACT:"Expert System" OR ABSTRACT:"Data Mining" OR ABSTRACT:"text mining" OR ABSTRACT:"Big Data" OR ABSTRACT:"Data Science" OR ABSTRACT:"Data Analytics" OR ABSTRACT:"Data driven" OR ABSTRACT:"Bayesian" OR ABSTRACT:"Bayes") AND (ABSTRACT:"Primary Health Care" OR ABSTRACT:"Primary Care" OR ABSTRACT:"general practitioner" OR ABSTRACT:"Family Practice" OR ABSTRACT:"General Practice" OR ABSTRACT:"primary healthcare" OR ABSTRACT:"Community Medicine" OR ABSTRACT:"Community Practice" OR ABSTRACT:"community physician" OR ABSTRACT:"community nursing") AND (SRC:PPR)
Epistemonikos database	(("Artificial Intelligence" OR "machine learning" OR "Artificial Intelligence" OR "Biological Ontologies" OR "Biological Ontology" OR "Computational Intelligence" OR "Computer Heuristics" OR "Computer Intelligence" OR "Computer Neural Network" OR "Computer Neural Networks" OR "Computer Reasoning" OR "Computer Vision System" OR "Computer Vision Systems" OR "Deep Learning" OR "Expert System" OR "Expert Systems" OR "Fuzzy Logic" OR "Hierarchical Learning" OR "Knowledge Base" OR "Knowledge Bases" OR "Machine Intelligence" OR "Machine Learning" OR "Natural Language Processing" OR "Robotic" OR "Robotics" OR "Supervised Machine Learning" OR "Support Vector Machine" OR "Support Vector Machines" OR "Unsupervised Machine Learning" OR "Knowledge Acquisition" OR "Knowledge Representation" OR "Neural Network" OR "Neural Networks" OR "Neural Network" OR "Data Mining" OR "Data Mining" OR "text mining" OR "Multifactor Dimensionality Reduction" OR "Big Data" OR "Big Data" OR "Data Science" OR "Data Science" OR "Data Sciences" OR "Data Analytics" OR "Data driven" OR "Bayesian Network" OR "Bayes Network" OR "Bayesian Networks" OR "Bayes Networks" OR "Bayesian Network" OR "Bayes Network" OR "Bayesian Networks" OR "Bayes Networks" OR "Bayes Theorem") AND ("Primary Health Care" OR "Primary Care Nursing" OR "general practitioner" OR "General Practitioners" OR "Family Practice" OR "General Practice" OR "primary healthcare" OR "Family Practice" OR "General

© 2024 Rakers MM et al. *JAMA Network Open*

Database	Query
	Practice" OR "primary care" OR "primary health care" OR "Family Physician" OR "Family Physicians" OR "Family Doctor" OR "Family Doctors" OR "General Practitioner" OR "General Practitioners" OR "General Practice Physician" OR "General Practice Physicians" OR "Primary Medical Care" OR "Family Healthcare" OR "Family Health Care" OR "Family Medicine" OR "Nurse Practitioner" OR "Nurse Practitioners" OR "Family Nurse" OR "Family Nurses" OR "Family Nursing" OR "Community Medicine" OR "Community Medicine" OR "Community Practice" OR "community physician" OR "community physicians" OR "community doctor" OR "community doctor" OR "community practitioner" OR "community practitioner" OR "community nurse" OR "community nurses" OR "community nursing"))
PsycINFO	TX(("Artificial Intelligence" OR "machine learning" OR "Artificial Intelligence" OR "Biological Ontologies" OR "Biological Ontology" OR "Computational Intelligence" OR "Computer Heuristics" OR "Computer Intelligence" OR "Computer Neural Network" OR "Computer Neural Networks" OR "Computer Reasoning" OR "Computer Vision System" OR "Computer Vision Systems" OR "Deep Learning" OR "Expert System" OR "Expert Systems" OR "Fuzzy Logic" OR "Hierarchical Learning" OR "Knowledge Base" OR "Knowledge Bases" OR "Machine Intelligence" OR "Machine Learning" OR "Natural Language Processing" OR "Robotic" OR "Robotics" OR "Supervised Machine Learning" OR "Support Vector Machine" OR "Support Vector Machines" OR "Unsupervised Machine Learning" OR "Knowledge Acquisition" OR "Knowledge Representation" OR "Neural Network" OR "Neural Networks" OR "Neural Network" OR "Data Mining" OR "Data Mining" OR "text mining" OR "Multifactor Dimensionality Reduction" OR "Big Data" OR "Big Data" OR "Data Science" OR "Data Science" OR "Data Sciences" OR "Data Analytics" OR "Data driven" OR "Bayesian Network" OR "Bayes Network" OR "Bayesian Networks" OR "Bayes Networks" OR "Bayesian Network" OR "Bayes Network" OR "Bayesian Networks" OR "Bayes Networks" OR "Bayes Theorem") AND ("Primary Health Care" OR "Primary Care Nursing" OR "general practitioner" OR "General Practitioners" OR "Family Practice" OR "General Practice" OR "primary healthcare" OR "Family Practice" OR "General Practice" OR "primary care" OR "primary health care" OR "Family Physician" OR "Family Physicians" OR "Family Doctor" OR "Family Doctors" OR "General Practitioner" OR "General Practitioners" OR "General Practice Physician" OR "General Practice Physicians" OR "Primary Medical Care" OR "Family Healthcare" OR "Family Health Care" OR "Family Medicine" OR "Nurse Practitioner" OR "Nurse Practitioners" OR "Family Nurse" OR "Family Nurses" OR "Family Nursing" OR "Community Medicine" OR "Community Medicine" OR "Community Practice" OR "community physician" OR "community physicians" OR "community doctor" OR "community doctor" OR "community practitioner" OR "community practitioner" OR "community nurse" OR "community nurses" OR "community nursing") AND ("Program Evaluation" OR "Evaluation Study" OR "Evaluation Studies as Topic" OR "Process Assessment" OR "Implementation Science" OR "Health Plan Implementation" OR "Pilot Projects" OR "Pilot Study" OR "Preliminary Data" OR "Adopting" OR "Adoption" OR "Assess" OR "Assessment" OR "Evaluation" OR "Evaluation studies" OR "Evaluation study" OR "Evaluations" OR "Implement" OR "Implementation" OR "pilot phase" OR "Pilot Studies" OR "Pilot Study" OR "Preliminary Data" OR "Process Assessments" OR "Process Assessment" OR "Process Measure" OR "Process Measures" OR "Program Appropriateness" OR "Program Assessments" OR "Program Assessment" OR "Program Effectiveness" OR "Program Evaluation" OR "Program Sustainability" OR "Programme Appropriateness" OR "Programme Assessment" OR "Programme Effectiveness" OR "Programme Evaluat*" OR "Programme Evaluation" OR "Programme Sustainability"))
GoogleScholar	"Artificial Intelligence"|"machine learning" "Primary Health Care"|"Primary Care"|"general practitioner"|"Family Practice"|"General Practice"|"primary healthcare" "Program

© 2024 Rakers MM et al. *JAMA Network Open*

Database	Query
	Evaluation"|"Evaluation"|"Assessment"|"Implementation"|"Pilot"|"Adoption"

© 2024 Rakers MM et al. *JAMA Network Open*

eAppendix 2 –Selection Process

Three reviewers (MMR, SK and MMvB) independently screened studies for inclusion and exclusion criteria based on title and abstract. All reviewers then screened the selected studies based on the full text. The reviewers discussed disagreements about the inclusion/exclusion of studies before being referred to an independent senior reviewer (HvO) until a consensus was reached. Summarising information about each predictive ML algorithm was generated by one reviewer (MMR). This included the year of publication, CE mark, or FDA approval, the authors' country of residence, the types of predictive ML algorithm (e.g., decision support, risk stratification, etc.), the study design, and the health condition addressed. This information was confirmed by the second reviewer (MMvB).

© 2024 Rakers MM et al. *JAMA Network Open*

eAppendix 3 – Online questionnaire for information from authors and commercial product owners

Towards improving access to evidence of prediction machine learning (ML) algorithms implemented in primary care: a scoping review

Summary

AI has the potential to revolutionize the healthcare sector. Especially in primary care, where the setting may have a unique position to benefit from AI since the general practitioners have fewer diagnostic instruments or tests to diagnose or refer their patients. However, despite many technological developments, only a handful of AI applications have been implemented into clinical practice. As a result, the evidence base for the clinical effectiveness of these techniques is still sparse and inconsistent, hampering the trust in and adoption of AI in clinical settings. It is our belief that valuable examples of current AI applications help understand how qualitative and reliable AI could improve the quality of healthcare. Moreover, their scientific validation and market transparency can contribute to the safe and well-considered implementation of AI software.

Therefore, we conducted a scoping review with the aim to identify (the quality and reliability of) implemented AI in primary care. Commissioned by the Dutch Ministry of Health, researchers have published a guideline for high-quality AI in healthcare to discuss good professional conduct in the development, testing, and implementation of AI in the medical sector. In the appendix of this email, we have added the six phases with corresponding questions to each phase. We did not find all the information of your AI-model regarding those phases and would like to invite you to fill in the boxes with the appropriate information (if available). In this way, we hope that together we will aid in the transition from AI development to clinical implementation and through this boost adoption of effective, safe, and responsible clinical AI in primary care.

Details AI-system

1. Brief description of your AI-system: name, purpose/objective of the system and primary care function. *

Phase 1: Preparation and verification of the data

Phase 1 is about drawing up, managing and executing a data management plan. In this plan, agreements and procedures are laid down regarding the collection of required (meta) data, the storage of this (meta) data and its accessibility.

2. Is there a data management plan available? *

- Yes, data management plan contains information about legal preconditions, data collection, metadata and data availability.
- Part of the data management plan is accessible.
- There is no data management plan.
- Unknown

3. Comment, clarification or explanation (access to publicly available reported evidence)

Phase 2: Development of the AI-model

Phase 2 covers the development of the AI-model. The model is the set of algorithm-specific data structures that, in combination with an algorithm, forms the AI-model. Furthermore, it is the result of analysis of the training data.

The steps in this phase are:
- Definition of target use
- Analysis and modelling steps
- Internal validation
- Robustness
- Size of the dataset for AI model development
- Reproducibility and replicability

Definition of target use

1. Which medical or health process the AI-system is intended for?

It is mandatory to clarify at least the following in the intended that has been recorded:

i) For which medical or health application the AI-model is intended (e.g. in which medical context, indication or target population) and who the envisaged end-user is (e.g. a specific specialization, primary care provider, or the patient, client or citizen himself);
ii) Which medical or healthcare process the AI-model intends to influence and what the expected benefit is compared to the current process (e.g. faster diagnosis, improved estimate of a person's prognosis, or indication for modification of a lifestyle habit);
iii) What the envisaged timing of the use of the AI-model or the prediction will be (e.g. upon admission to the hospital or Intensive Care Unit, at the time of receiving a cancer diagnosis, upon referral for a CT scan, or when symptoms or complaints are observed, or when monitoring blood sugar levels);
iv) Whether this is a diagnostic, prognostic, monitoring, screening or other type of healthcare application;

2. Description of the origin of the dataset(s) for model development, data collection design, measurement and registration procedures.

4. Has the target been defined and reported? *

- Yes, all of these requirements in this phase are included in the publication about development of the AI-model.
- Part of these requirements in this phase are included in the publication about development of the AI-model.
- Target use has not been defined and reported.
- Unknown

5. Comment, clarification or explanation (access to publicly available reported evidence)

© 2024 Rakers MM et al. *JAMA Network Open*

6. Has the origin of the dataset been described? *

Yes, description of the origin of the dataset(s) for model development, data collection design, measurement and registration procedures have been mentioned.

There is some information about the origin of the dataset, but is has not been reported structurally.

There is no information about the origin of the dataset.

Unknown

7. Comment, clarification or explanation

Analysis and modeling steps

It is mandatory for the developer of the model to record all analysis and model development steps. This includes all preparatory steps (e.g. initial data analysis10, feature engineering), modelling technique used (e.g. neural network, random forest, time to event, logistic regression), all modelling steps (e.g. model selection, tuning, (re)calibration).

8. Have all modeling steps been mentioned? *

Yes, all of the steps in this phase are included in a publication/document about development of the AI-model.

Part of these steps in this phase are included in a publication/document about development of the AI-model.

Modeling steps have not been reported.

Unknown

© 2024 Rakers MM et al. *JAMA Network Open*

9. Comment, clarification or explanation

Internal validation

Internal validation is an important part of the process of development of the AI-model. The aim of
the internal validation is to quantify realistic estimates of the model performance of the AI-model.
An adequate estimator of the model performance (e.g. the C(oncordance) statistic and calibration curve) can differ between types of applications and endpoints (e.g. binary, multi-category, time-to-event).

10. Has the internal validation process been clearly described? *

- Yes, the AI-model internal validation has been described and was strictly separated from model development steps
- There is information about the internal validation process, but it is not strictly seperated from model development steps.
- There is no information about the internal validation of the AI-model.
- Unknown

11. Comment, clarification or explanation (access to publicly available reported evidence)

© 2024 Rakers MM et al. *JAMA Network Open*

Robustness

During the development of the AI-model, it is mandatory to examine the technical robustness of the model and record the findings in a transparent manner, at least for those models that are used in the external validation (phase 3).

Various sensitivity analyses are recommended in order to study the robustness. This can include analyses of the:
- Architectural robustness: repetition of the analysis steps on the same data results in a model that does not deviate significantly from the original model.
- Consistency of model performance: repetition of the analysis steps on the same data results in models with performance that does not deviate much from the performance of the original AI-model.
- Adversarial robustness: the effect of a (deliberate) disruption on the input variables of the model on the performance and/or architecture.
- Domain shift and outliers: the effect of any outliers in the data and/or deliberate changes to the data set (e.g. deliberate inclusion or exclusion of certain groups) on the model performance and/or the architecture (e.g. outlier rejection analysis). Also refer to phase 4 and 6 for additional activities

12. Has the robustness of the model been investigated? *

- Yes, an adequate sensitivity analysis has been performed
- There is some information about the sensitivity analysis, but is has not been described clearly.
- There is no information about an adequate sensitivity analysis.
- Unknown

13. Comment, clarification or explanation (access to publicly available reported evidence)

© 2024 Rakers MM et al. *JAMA Network Open*

Size of the dataset for AI-model development

The starting point for choosing the size of the dataset within model development is: the bigger, the better. However, this starting point must be weighed against medical-ethical considerations.

14. Has the size needed for AI-model development of the dataset been calculated? *

- Yes, a calculation of the size of the dataset has been described (e.g. a priori or a posteriori method has been used to evaluate the minimal size of the dataset).
- There is information about the size of the dataset, but little information has been provided about the method of calculating this size.
- There is no information about the size of the dataset and its calculations.
- Unknown

15. Comment, clarification or explanation (access to publicly available reported evidence)

Reproducibilty and replicability

It is mandatory for all analysis steps and internal validation steps and the analysis of technical robustness to be logged accurately in order to guarantee the reproducibility (i.e. the ability to repeat the development using different data).

© 2024 Rakers MM et al. *JAMA Network Open*

16. Have the steps of the internal validation been reported transparently? *

- Yes, steps, procedures, and used data have been described transparantly.
- Part of the steps of the internal validation has been reported transparently.
- There is no record of the steps of internal validation accesible.
- Unknown

17. Comment, clarification or explanation (access to publicly available reported evidence)

Phase 3: Validation of the AI-model

Phase 3 consist of (external) validation of the AI-model developed in phase 2. External validation refers to the evaluation of the AI-model with data that has not been used for development in phase 2. Here, we appoint a difference between the evaluation of the statistical or predictive value, the evaluation of the (added) value compared to current care practice and the evaluation of fairness and algorithmic bias. External validation does explicitly not mean: the (re-)training or (re-)tuning of a model.

Evaluation of (statisical) characteristics of the AI-model

1. The dataset used for external validation is different to the development dataset, and is representative for the target population and context.
2. It is mandatory for an exact description of the origin of the data (e.g. time and place), the method of data collection (e.g. consecutive patients), measurement and registration procedures, any selections and inclusion and exclusion criteria to be recorded in the data management plan in order to define the context of the characteristics.

18. Is the dataset used for external validation clearly described in the documentation/publication? *

- Yes, this has been clearly described.
- Partly described in the documentation/publication.
- There is no documentation/publication about the external validation.
- Unknown

19. Comment, clarification or explanation (access to publicly available reported evidence)

20. Has the origin of the dataset been described? *

.

- Yes, description of the origin of the dataset(s) (other than the one used for internal validation), data collection design, measurement and registration procedures have been mentioned.
- There is some information about the origin of the dataset, but is has not been reported structurally.
- There is no information about the origin of the dataset.
- Unknown

21. Comment, clarification or explanation (access to publicly available reported evidence)

Fairness and algoritmic bias

The external validation of the AI-model model should look beyond the model performance and medical value. The evaluation of fairness and bias is also very important.

22. Are fairness of the algorithm and the presence of different bias analysed and recorded? *

- Yes, the fairness and biases have been clearly described.
- Part of the fairness and biases have been described.
- There is no information about the fairness and algoritmic bias.
- Unknown

23. Comment, clarification or explanation (access to publicly available reported evidence)

© 2024 Rakers MM et al. *JAMA Network Open*

Determining the outcome variable

The accurate determination of the outcome that is to be predicted in the external validation data set is an important factor for the validity of the statistical model performance and the medical value. There are many situations in medicine where no gold standard is available for the measurement of the outcome variable (e.g. for some diagnoses, classifications or cause specific mortality), which potentially result in misclassification of the outcome variable. Therefore, the term "reference standard" is often used. In some situations, evaluation by an expert or group of experts is required to arrive at a decision per case (e.g. the assessment of a tumour on a CT scan).
It is mandatory to perform so-called labelling of outcomes in the data set for external validation as accurately as possible in this phase and to record and justify this process as transparently as possible.

24. Has the definition and process of the labels transparently been described? *

- Yes, outcome variables have been well described and considered.
- There is some information about the outcome variables, but the considirations are not transparantly documented.
- There is no information about the determination of the outcome variables.
- Unknown

25. Comment, clarification or explanation (access to publicly available reported evidence)

Reproducibilty and replicability

As for phase 2, reproducibility and replicability are important guiding principles for external validation of the AI-model.
In order to guarantee reproducibility (i.e. repeat of the external validation using different data), it is mandatory to log the process that was followed and the data that was used for external validation in a complete and transparent manner, even in the case of negative results

26. Have the steps of the external validation been reported transparently? *

- Yes, steps, procedures and used data have been described transparantly.
- Part of the steps, procedures and used data have been described.
- There is no record of the steps of external validation accesible.
- Unknown

27. Comment, clarification or explanation (access to publicly available reported evidence)

Size of the dataset for external validation

The starting point for choosing the size of the dataset within model development is: the bigger, the better. The larger the data set, the more precise the estimates can be used for statistical and medical evaluation and testing of algorithmic bias.

28. Has the size of the dataset needed for validation been calculated? *

- Yes, a calculating of the size of the dataset has been described (e.g. a priori or a posteriori method has been used to evaluate the minimal size of the dataset).
- There is information about the size of the dataset, but little information has been provided about the method of calculating this size.
- There is no information about the size of the dataset and its calculations.
- Unknown

© 2024 Rakers MM et al. *JAMA Network Open*

29. Comment, clarification or explanation (access to publicly available reported evidence)

Phase 4: Development of the necessary software application

Phase 4 covers the development of the software around the AI-model by the manufacturer. That is, the design, development, user testing and associated system requirements for the software

Explainability, transparency, design and information

1. Explainable model
2. Complex (black box) algorithm

The outcomes of the AI-model will be presented in the software in a transparent and explainable manner. The presentation of the outcomes of the AI-model in the software distinguishes between an inherently explainable and a complex model.

30. What type of model is used? *

- Explainable model *Ga naar vraag 32*
- Complex (black box) algorithm *Ga naar vraag 34*

31. Comment, clarification or explanation (access to publicly available reported evidence)

© 2024 Rakers MM et al. *JAMA Network Open*

© 2024 Rakers MM et al. *JAMA Network Open*

Explainable model

In the case of an inherently explainable model, it is mandatory that the manufacturer discloses information about the interpretation of the model and the model predictions for the intended end-users.

32. Are the results of the AI-model presented in the software in a transparant and explainable manner? *

- Yes, information on the interpretation of the AI-model are available for the intended end users in the presentation.
- There is information about the interpretation of the outcomes of the model, but is it not provided to all endusers.
- There is no explanation of the outcomes being formulated by the AI-model.
- Unknown

33. Comment, clarification or explanation (access to publicly available reported evidence)

Ga naar vraag 36

Complex (black box) algorithm

In the case of a complex model (e.g. an algorithm based on deep learning), the relationship between input variables and predicted outcomes is so complex that it is no longer possible to comprehend this (so-called "black box" algorithms). Extra attention should be paid to post-hoc information and interpretation of the model in the presentation of the model by the Software.

It is mandatory to substantiate the following aspects of complex models: 1) why an explainable model was not used and 2) if opting for a post-hoc explanation, why this is appropriate for the model and the intended end-user.

34. Are the results of the AI-model presented in the software in a transparant and explainable manner? *

- Yes, explanation about 1) why the model isn't explainable, or 2) why a post-hoc explanation was chosen
- There is information about the interpretation of the outcomes of the model, but is it not provided to all endusers.
- There is no explanation of the outcomes being formulated by the AI-model.
- Unknown

35. Comment, clarification or explanation (access to publicly available reported evidence)

Phase 4 Development of the necessary software application

Testing

Existing software standards and regulations (e.g. IEC, ISO, FDA)

36. Are the required standards and regulations met (if applicable)? *

- Yes, the software disposes of the required standards and regulations (if applicable).
- Part of the required standards and regulation have been met/described.
- The software doesn't meet the required standards and regulations.
- Unknown
- AI-model isn't in this phase yet

37. Comment, clarification or explanation (access to publicly available reported evidence)

Phase 5: Impact assessment of the AI-model in combination with the software

Phase 5 covers the determination of the impact or added value of the use of the AI-model as part
of the software on the envisaged medical practice or context, the medical treatment and the health outcomes respectively for the intended group (e.g. the patient, client or citizen). A Health technology assessment is also performed during this phase. The manufacturer (or the developing care organization, in the event of internal development) is responsible for determining the impact and added value. Still, this process is generally performed in collaboration with developers, care organizations and end-users.

Impact assessment

1. Expected effects on possible and relevant (health and process) outcomes.

In addition to the intended use that has been recorded, it is mandatory to record in more detail what the expected effects of the use of the AI-model are on possibly relevant (health and process) outcomes (in other words, define the intended use of the AI-model).

2. Risk assessment

It is mandatory to perform a risk assessment to gain insight into the potential risks of the use of the AI-model in daily medical practice.
This includes the expected unintended decisions and effects in the entire care process (refer to Section 5.1.1) and reasonably foreseeable incorrect use;
It is mandatory to create an inventory of the potential undesirable effects (risks) of implementation of the AI-model in the care process per component of the care process in the risk assessment, in close cooperation with the stakeholders (e.g. end-users and patients).

It is mandatory to select and implement risk-mitigating measures for the risks identified during the risk assessment.
It is mandatory to include any sources of uncertainty as listed in 5.3.1a in the risk assessment.
It is mandatory to incorporate any risks identified in the risk assessment in the outcomes of the empirical study (refer to Section 5.1.4).

3. Human-machine interaction

The effectiveness of the interaction of the end-user with the software is of great importance to the impact of the AI-model software.
It is mandatory to ensure that the AI-model software interfaces with the current medical care processes and accompanying medical decision making as seamlessly as possible before an empirical study is performed (refer to Figure 2 in Section 5.1.1).
It is mandatory to involve several end-users in the local implementation team in order to achieve this (also refer to phase 6 for the further composition of an implementation team). In addition, it is mandatory to create an inventory of the expected changes in the care context (e.g. changes in the work process) caused by the software, preferably in consultation with the intended user and patient, client or citizen.

4. (Comparative) study design

In order to ensure a valid quantification of the added benefit of the implementation of an AI-model in the context of the daily medical practices, it is mandatory to perform a comparative
study, in which the (desirable and undesirable) effects of the use of the AI-model (refer to Section 5.1.1) are compared to a similar context, in which similar standard care is performed, without the use of the AI-model.

© 2024 Rakers MM et al. *JAMA Network Open*

38. Has an impact assessment been conducted? *

- Yes, an impact assessment has been conducted.
- Part of an impact assessment has been conducted.
- No impact assessment has been conducted.
- Unknown
- AI-model isn't in this phase yet

39. Comment, clarification or explanation (access to publicly available reported evidence)

40. Has a risk assessment been carried out to identify the potential risks using the AI-model in daily medical practice. *

- Yes, a risk assesment has been clearly described.
- No risk assesment has been described.
- Unknown
- AI-model isn't in this phase yet

41. Comment, clarification or explanation

© 2024 Rakers MM et al. *JAMA Network Open*

42. Are all end-users involved in the implementation team? And does the AI model fit the current health processes and associated medical decision making? *

.

- Yes, all end-users are involved in the implementation team. The the AI model fits the current health processes and is associated with the medical decision making?
- End-users are only involved at a later stage. Part of the suitability of the AI model in current health processes and decision making have been described.
- End-users were not involved in the implementation team.
- Unknown
- AI-model isn't in this phase yet

43. Comment, clarification or explanation (access to publicly available reported evidence)

44. The choice for a comparative study design must be carried out and substantiated (including the population/context in which it is carried out). *

- Yes, a comparative study did take place and if not, there was a well-founded reason to use an other study design.
- No comparative study design was used, and the reason to provide an other study design was not clear and well-founded.
- No comparative study design was used, and the reason to provide an other study design wasn't mentioned either.
- Unknown
- AI-model isn't in this phase yet

45. Comment, clarification or explanation (access to publicly available reported evidence)

Health Technology Assessment (HTA)

It is strongly recommended that phase 5 includes a model-based impact analysis, or a model-based Technology Assessment (HTA).
In other words, a mathematical model (e.g. a Markov model) is used to provide an objective analysis of the expected costs and benefits (added value) of the introduction of the AI-model in
the medical practice compared to the current standard of care as benchmark or control. The result of such an HTA will become increasingly important in the approval of digital healthcare in the Netherlands and the EU. If reimbursement is essential, an appropriate HTA is thus required to be eligible for such reimbursement – or conditional reimbursement – of the implementation. T

46. Has a HTA been performed? *

- Yes, a HTA was performed.
- No, a HTA was not performed.
- Unknown
- AI-model isn't in this phase yet

47. Comment, clarification or explanation (access to publicly available reported evidence)

© 2024 Rakers MM et al. *JAMA Network Open*

© 2024 Rakers MM et al. *JAMA Network Open*

Phase 6: Implementation and use of the AI-model with software in daily practice

Phase 6 covers the implementation and use of the AI-model in clinical practice. Central themes in this phase are implementation, monitoring and education.

Implementation plan

When an AI-model is implemented and applied within a care organisation, it is mandatory that the care organisation drafts an implementation plan.
An implementation plan includes both the technical implementation of the AI-model and the software in the existing (IT) infrastructure and the embedding of the use of the AI-model in existing work processes. Please refer to the Covenant on Medical Technology9 and the Guideline New Interventions in Clinical Practice for a general guideline for implementation.
As part of the local implementation process, it is mandatory to evaluate the reliability and applicability of the AI-model by means of an assessment of (the results of) previous studies performed as part of phase 3 and 5.
If these results provide an inadequate indication of the reliability and applicability of the AI-model
within the local context, additional validation of the AI-model can be performed (also refer to phase 3).
In addition, it is mandatory to introduce the AI-model – and the accompanying work process in which the AI-model is used – into the care process in a controlled manner, for example, in the form of a pilot, run-in period or by means of parallel implementation of the AI-model alongside the traditional care process.
It is mandatory to perform a prospective risk inventory (PRI) to gain insight into the potential risks of the use of the AI-model in daily medical practice.

48. Is there a implemenationplan available that covers all the required components * as described above?

- ◯ Yes, implementation plan meets the required components of an implementation plan.
- ◯ There is information about the implementation plan, but not all components have been described.
- ◯ No implemenation plan with the required components is available.
- ◯ Unknown
- ◯ AI-model isn't in this phase yet

49. Comment, clarification or explanation (access to publicly available reported evidence)

Monitoring

The manufacturer and the healthcare institution monitor for technical errors in the AI-model and associated software, for misuse, for fairness and unexpected side effects of ordinary use of the software application in daily practice.

50. Is there a monitoring plan available that covers all the required components as described above? *

- Yes, monitoring plan meets the required components of a monitoring plan.
- There is information about the monitoring plan, but not all components have been mentioned.
- No monitoringplan with the required components is available.
- Unknown
- AI-model isn't in this phase yet

51. Comment, clarification or explanation (access to publicly available reported evidence)

© 2024 Rakers MM et al. *JAMA Network Open*

© 2024 Rakers MM et al. *JAMA Network Open*

Education

It is mandatory that the end-user (e.g. a patient or the care provider) has access to information about the topics described in Box 6.1, to be supplied by the developer or manufacturer.
If the end-user is a care provider, it is mandatory that the care provider has access to education about the topics described in Box 6.2.

52. Are there education modules accessible for end users and/or healthcare institutions? *

Markeer slechts één ovaal.

- Yes, there is eduation about the AI-model/software is accessible for end users and/or healthcare institutions.
- Partial support of eduation about the AI-model/software is accessible for end users and/or healthcare institutions.
- No education accessible.
- Unknown
- AI-model isn't in this phase yet

53. Comment, clarification or explanation (access to publicly available reported evidence)

© 2024 Rakers MM et al. *JAMA Network Open*

eTable 1 – The evidence requirements established per life cycle phase as described in the Dutch AIPA guideline

			Phase 1: Collection and management of		Phase 2: Development of the AI-model								Phase 3: Validation of the AI-model					
**CRITERIA**			Datamanagementplan		Definement target use		Analysis and modeling steps	Internal validation	Robustness	Size of the dataset for AI-model development	Reproducibility and replicability		Evaluation statistic properties the AI-model		Fairness and algoritmic bias	Determining the outcome variable	Reproducibility and replicability	Size of the dataset for external validation
**EXPLANATION (only requirements are obtained from the guideline)**			Phase 1 is about drawing up, managing and executing a data management plan. In this plan, agreements and procedures are laid down regarding the collection of required (meta) data, the storage of this (meta) data and its accessibility.		Definement and record of a clear definition of the intended use of the AI model.	Description of the origin of the dataset(s) for model development, data collection design, measurement and registration procedures. If there	Recording of all preparation steps, modeling technique used, all modeling steps.	Internal validation is a key step of the development process and refers to the realistic estimation of predictive performance in a separate group.	Sensitivity analysis	The starting point for choosing the size of the dataset within model development is: the bigger, the better. However, this starting point must be weighed against medical-ethical considerations.	Steps and procedures of internal validation are documented transparently		The dataset used for external validation is different to the development dataset, and is representative for the target population and context.	Description of the origin of the dataset(s) for model development, data collection design, measurement and registration procedures. If there are major differences between development and external validation	Fairness of the algorithm and the presence of different bias must be investigated.	Accurately determining the (predictable) outcome in the external validation dataset (labeling)	Steps and procedures of external validation are documented transparently.	The starting point for choosing the size of the dataset within model development is: the bigger, the better. The larger the data set, the more precise the estimates can be used for statistical and medical evaluation and testing of algorithmic
**QUESTION**	**Color coding**	**Values**			Has the target been defined and reported?	Has the origin of the dataset been described?	Have analysis and modeling steps been mentioned?	Has the internal validation process been clearly	Has the robustness of the model been investigated?	Has the size needed for AI-model development of the dataset	Have the steps of the internal validation been		Is this clearly described in the external validation documentati	Has the origin of the dataset been described?	Are fairness of the algorithm and the presence of different	Has the definition of the labels transparantly been	Have the steps of the external validation been	Has the size of the dataset needed for validation been
**No information / unknown**		0	Unknown		Unknown	Unknown	Unknown	Unknown	Unknown	Unknown	Unknown		Unknown	Unknown	Unknown	Unknown	Unknown	Unknown
**Meets the criteria**		2	Yes, datamanagementplan contains information about legal preconditions, data collection, metadata and data availability.		Yes, all of these requirements in this phase are included in a report about development of the AI-model.	Yes, description of the origin of the dataset(s) for model development, data collection design, measurement and registration procedures have been mentioned.	Yes, all of the steps in this phase are included in a report about development of the AI-model.	Yes, the AI-model internal validation has been described and was strictly separated from model development steps	Yes, an adequate sensitivity analysis has been performed	Yes, a calculating of the size of the dataset has been described (e.g. a priori or a posteriori method has been used to evaluate the minimal size of the dataset).	Yes, steps, procedures and used data have been described transparantly.		Yes, this has been clearly described.	Yes, description of the origin of the dataset(s) (other than the one used for internal validation), data collection design, measurement and registration procedures have been mentioned.	Yes, the fairness and biases have been clearly described.	Yes, outcome variables have been well described and considered.	Yes, steps, procedures and used data have been described transparantly.	Yes, a calculating of the size of the dataset has been described (e.g. a priori or a posteriori method has been used to evaluate the minimal size of the dataset).
**Partly meets the criteria**		1	Part of the datamangementplan is accessible.		Part of these requirements in this phase are included in a report about development of the AI-model.	There is some information about the origin of the dataset, but is has not been reported structurally.	Part of these steps in this phase are included in a report about development of the AI-model.	There is information about the internal validation process, but it is not strictly seperated from model development steps.	There is some information about the sensitivity analysis, but is has not been described clearly.	There is information about the size of the dataset, but little information has been provided about the method of calculating	Part of the steps of the internal validation has been reported transparantly.		Partly described in the documentation/publication.	There is some information about the origin of the dataset, but is has not been reported structurally.	Part of the fairness and biases have been described.	There is information about the outcome variables, but the considerations are not transparantly documented.	Part of the steps, procedures and used data have been described.	There is information about the size of the dataset, but little information has been provided about the method of calculating

			Phase 4: Development of the necessary software application			Phase 5: Impact assessment of the AI-model in combination with the software						Phase 6: Implementation and use of the AI-model with software in daily practice		
**CRITERIA**			Explainability, transparency, design and information	Testing		Impact assessment				Health Technology Assessment (HTA)		Implementationplan	Monitoring	Education
**EXPLANATION (only requirements are obtained from the guideline)**			Explainable model	Existing software standards and regulations (e.g. IEC, ISO, FDA)		Expected effects on possible and relevant (health and process) outcomes	Risk assessment	Human-machine interaction	(Comparative) study design	Analysis of anticipated costs and benefits.		Consist of: a technical implementation plan, accompanying work process, prospective risk-inventarisation, and drafting of an implementation-team	The manufacturer and the healthcare institution monitor for technical errors in the AI-model and associated software, for misuse, for fairness and unexpected side effects of ordinary use of the software application in	Education about the AI-model/software for end users and healthcare institutions
**QUESTION**	**Color coding**	**Values**	Are the results of the AI-model presented in the software	Are the required standards and regulations		Have the expected decisions by the end-user, and the	Has a risk assessment been carried out to identify the potential	Are all endusers involved in the implementatio	The choice for a comparative study design must be	Is there a HTA done?		Is there a implemenationplan available that cover all the	Is there a monitoringsplan available that cover all the required	Are there education modules accessible for end users
**No information / unknown**		0	Unknown	Unknown		Unknown	Unknown	Unknown	Unknown	Unknown		Unknown	Unknown	Unknown
**Meets the criteria**		2	Yes, explanation about 1) why the model isn't explainable, or 2) why a post-hoc explanation was chosen	Yes, the software disposes of the required standards and regulations (if necessary).		Yes, the expected decisions by the end-user, and the expected effects and consequences of the decisions on later (health) outcomes of the patient, client, citizen and/or on the local care context or on society have been described.	Yes, a risk assesment has been clearly described.	Yes, all end users are involved in the implementation team. The AI-model fits the current health processes and is associated with the medical decision making.	Yes, a comparative study did take place and if not, there was a well-founded reason to use an other study design.	Yes, a HTA was done.		Yes, implementationplan meets the required components of an implementationplan.	Yes, monitoringsplan meets the required components of a monitoringplan.	Yes, there is eduation about the AI-model/software is accessible for end users and/or healthcare institutions.
**Partly meets the criteria**		1	There is information about the interpretation of the outcomes of the model, but is it not provided to all endusers.	Part of the required standards and regulation have been met/described.		Part of the expected decisions by the end-user, and the expected effects and consequences of the decisions on later (health) outcomes of	Part of a risk assessment has been conducted.	Part of the end users are involved. Part of the suitability of the AI-model in current health processes and decision making have been	No comparative study design was used, and the reason to provide an other study design was not clear and well-founded.	No proper HTA was performed, but cost calculations were done.		There is information about the implementationplan, but not all components have been described.	There is information about the monitoringsplan, but not all components have been mentioned.	Partial support of eduation about the AI-model/software is accessible for end users and/or healthcare institutions.

© 2024 Rakers MM et al. *JAMA Network Open*

eFigure – Flowchart of literature inclusion for assessment of the six phases

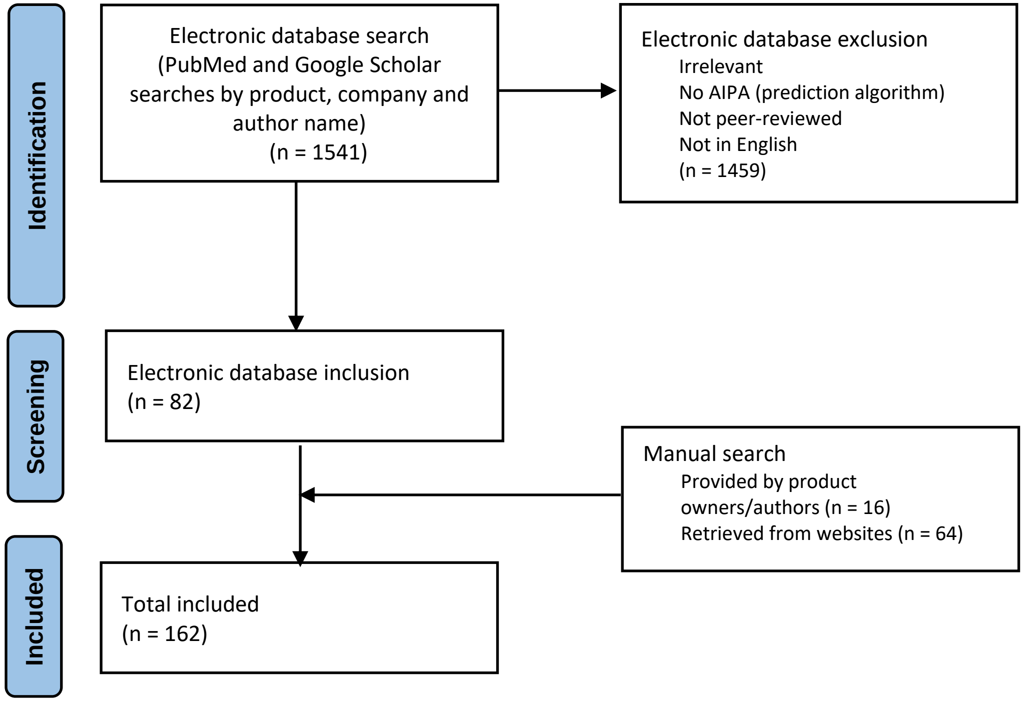

eTable 2 – Overview of publication characteristics per predictive ML algorithm

Predictive ML algorithm	Product	Publication	doi	source	first author	year	source	center corresponding author
AIFRED (D. Benrimoh et al., 2021)	-	Aifred Health, a Deep Learning Powered Clinical Decision Support System for Mental Health	10.1007/978-3-319-94042-7_13	electronic database	D. Benrimoh	2018	The NIPS '17 Competition: Building Intelligent Systems	University College London, London, UK
AIFRED (D. Benrimoh et al., 2021)	-	Analysis of Features Selected by a Deep Learning Model for Differential Treatment Selection in Depression	10.3389%2Ffrai.2019.00031	electronic database	J. Mehltretter	2019	Frontiers in Artificial Intelligence	University of Southern California, Los Angeles, USA
AIFRED (D. Benrimoh et al., 2021)	-	Differential Treatment Benefit Prediction for Treatment Selection in Depression: A Deep Learning Analysis of STAR*D and CO-MED Data	10.1162/cpsy_a_00029	electronic database	J. Mehltretter	2020	Computational Psychiatry	University of Southern California, Los Angeles, USA
AIFRED (D. Benrimoh et al., 2021)	-	Evaluating the Clinical Feasibility of an Artificial Intelligence–Powered, Web-Based Clinical Decision Support System for the Treatment of Depression in Adults: Longitudinal Feasibility Study	10.2196/31862	electronic database	C. Popescu	2021	JMIR Formative Research	Aifred Health Inc., Montreal, Canada
AIFRED (D. Benrimoh et al., 2021)	-	Evaluating the Usability and Impact of an Artificial Intelligence-Powered Clinical Decision Support System for Depression Treatment	10.1016/j.biopsych.2020.02.451	electronic database	M. Tanguay-Sela	2020	Biological Psychiatry	Aifred Health Inc., Montreal, Canada
AIFRED (D. Benrimoh et al., 2021)	-	Evaluating the perceived utility of an artificial intelligence-powered clinical decision support system for depression treatment using a simulation center	10.1016/j.psychres.2021.114336	electronic database	M. Tanguay-Sela	2021	Psychiatry Research	Aifred Health Inc., Montreal, Canada
AIFRED (D. Benrimoh et al., 2021)	-	Using a simulation centre to evaluate preliminary acceptability and impact of an artificial intelligence-powered clinical decision support system for depression treatment on the physician-patient interaction	10.1192/bjo.2020.127	electronic database	D. Benrimoh	2021	BJPsych Open	University College London, London, UK

© 2024 Rakers MM et al. *JAMA Network Open*

Predictive ML algorithm	Product	Publication	doi	source	first author	year	source	center corresponding author
AIFRED (D. Benrimoh et al., 2021)	-	A Mixed-Methods Feasibility Study of a Novel AI-Enabled, Web-Based, Clinical Decision Support System for the Treatment of Major Depression in Adults	10.1101/2022.01.14.22269265	electronic database	S. Qassim	2022	BMJ Yale	University of Waterloo, Canada
No name (EW Breithart et al., 2020)	-	Improved patient satisfaction and diagnostic accuracy in skin diseases with a Visual Clinical Decision Support System—A feasibility study with general practitioners	10.1371/journal.pone.0235410	electronic database	Breithart	2020	PLoS One	Association of Dermatological Prevention (ADP), Hamburg, Germany
A-GPS (HY Seol et al., 2021)	-	Assessing socioeconomic bias in machine learning algorithms in health care: a case study of the HOUSES index	10.1093/jamia/ocac052	electronic database	Young J Juhn	2022	Journal of the American Medical Informatics Association	Mayo Clinic, USA
A-GPS (HY Seol et al., 2021)	-	A Technical Performance Study and Proposed Systematic and Comprehensive Evaluation of an ML-based CDS Solution for Pediatric Asthma	Abstract	author	Young J Juhn	2022	AMAI 2022 Informatics Summit	Mayo Clinic, USA
A-GPS (HY Seol et al., 2021)	-	Artificial intelligence-assisted clinical decision support for childhood asthma management: A randomized clinical trial	10.1371/journal.pone.0255261	electronic database	Hee Yun Seol	2021	PLoS One	Mayo Clinic, USA
No name (E Frontoni et al., 2020)	-	A Decision Support System for Diabetes Chronic Care Models Based on General Practitioner Engagement and EHR Data Sharing	10.1109/JTEHM.2020.3031107	electronic database	Emanuele Frontoni	2020	IEEE Journal of Translational Engineering in Health and Medicine	University of Politecnica, Italy
No name (E Frontoni et al., 2020)	-	A Shared Decision-Making System for Diabetes Medication Choice Utilizing Electronic Health Record Data	10.1109/JBHI.2016.2614991	electronic database	Yu Wang	2017	IEEE Journal of Biomedical and Health Informatics	Zhejiang University, Hangzhou, China

Predictive ML algorithm	Product	Publication	doi	source	first author	year	source	center corresponding author
No name (E Frontoni et al., 2020)	-	Development of a Service-Oriented Sharable Clinical Decision Support System Based on Ontology for Chronic Disease	10.3233/978-1-61499-830-3-1153	electronic database	Yong Shang	2017	Studies in Health Technology and Informatics	Zhejiang University, Hangzhou, China
No name (P Bachtiger et al., 2022)	-	Screening for cardiac contractile dysfunction using an artificial intelligence–enabled electrocardiogram	10.1038/s41591-018-0240-2	electronic database	Zachi I. Attia	2019	Nature Medicine	Mayo Clinic, Rochester, USA
No name (P Bachtiger et al., 2022)	-	Point-of-care screening for heart failure with reduced ejection fraction using artificial intelligence during ECG-enabled stethoscope examination in London, UK: a prospective, observational, multicentre study	10.1016/S2589-7500(21)00256-9	electronic database	Bachtiger	2022	Lancet Digital Health	Imperial College Healthcare NHS Trust, London, UK
No name (Y Kanagasingam et al., 2018) Retinopathy in Primary Care"	-	Lappeenranta University of Technology Diabetic Retinopathy Database and Evaluation Protocol	-	electronic database	Tomi Kauppi	2009	Machine Vision and Pattern Recognition Laboratory	Lappeenranta University of Technology
No name (Y Kanagasingam et al., 2018) Retinopathy in Primary Care"	-	Kaggle Diabetic Retinopathy Database.	-	electronic database	-	-	-	-
No name (Y Kanagasingam et al., 2018) Retinopathy in Primary Care"	-	Evaluation of Artificial Intelligence–Based Grading of Diabetic Retinopathy in Primary Care	10.1001/jamanetworkopen.2018.2665	electronic database	Kanagasingam	2019	JAMA Network Open	Australian e-Health Research Centre, Commonwealth Scientific and Industrial Research Organisation, Perth, Western Australia,

© 2024 Rakers MM et al. *JAMA Network Open*

**Predictive ML algorithm**	**Product**	**Publication**	**doi**	**source**	**first author**	**year**	**source**	**center corresponding author**
								Australia
EyeArt (J Liu et al., 2021)	-	Development and Validation of a Deep Learning Algorithm for Detection of Diabetic Retinopathy in Retinal Fundus Photographs	10.1001/jama.2016.17216	electronic database	Varun Gulshan	2016	Innovation in Health Care Deliery	Google Inc, Mountain View, California
EyeArt (J Liu et al., 2021)	-	Automated Diabetic Retinopathy Image Assessment Software: Diagnostic Accuracy and Cost-Effectiveness Compared with Human Graders	10.1016/j.ophtha.2016.11.014	electronic database	Adnan Tufail	2016	Ophthalmology	Moorfields Eye Hospital, London, UK
EyeArt (J Liu et al., 2021)	-	An observational study to assess if automated diabetic retinopathy image assessment software can replace one or more steps of manual imaging grading and to determine their cost-effectiveness.	10.3310/hta20920	electronic database	Tufail A.	2016	Health Technology Assessment	University College London Institute of Ophthalmology.
EyeArt (J Liu et al., 2021)	-	The Value of Automated Diabetic Retinopathy Screening with the EyeArt System: A Study of More Than 100,000 Consecutive Encounters from People with Diabetes	10.1089%2Fdia.2019.0164	electronic database	Malavika Bhaskaranand	2019	Diabetes Technology & Therapeutics	Eyenuk California
EyeArt (J Liu et al., 2021)	-	Performance of a Deep-Learning Algorithm vs Manual Grading for Detecting Diabetic Retinopathy in India	10.1001/jamaophthalmol.2019.2004	electronic database	Varun Gulshan	2019	JAMA Ophthalmology	Google Inc, Mountain View, California
EyeArt (J Liu et al., 2021)	-	Pivotal Evaluation of an Artificial Intelligence System for Autonomous Detection of Referrable and Vision-Threatening Diabetic Retinopathy	10.1001/jamanetworkopen.2021.34254	website	Eli Ipp	2021	JAMA Network Open	Eyenuk California
EyeArt (J Liu et al., 2021)	-	Diabetic Retinopathy Screening with Automated Retinal Image Analysis in a Primary Care Setting Improves Adherence to Ophthalmic Care	10.1016/j.oret.2020.06.016	electronic database	Liu	2021	Ophthalmology Retina	Department of Ophthalmology and Visual Sciences, Washington University School of Medicine, St.

© 2024 Rakers MM et al. *JAMA Network Open*

**Predictive ML algorithm**	**Product**	**Publication**	**doi**	**source**	**first author**	**year**	**source**	**center corresponding author**
								Louis, Missouri
No name (J Long et al., 2016)	-	An Observational Study to Evaluate the Usability and Intent to Adopt an Artificial Intelligence-Powered Medication Reconciliation Tool	10.2196/ijmr.5462	electronic database	Long	2016	Interactive Journal of Medical Research	Texas State University , San Marcos, TX, US
No name (S Romero-Brufau et al., 2020)	-	A lesson in implementation: A pre-post study of providers' experience with artificial intelligence-based clinical decision support	10.1016/j.ijmedinf.2019.104072	electronic database	Romero-Brufau	2020	International Journal of Medecal Informatics	Mayo Clinic, Minnesota, United States
No name (HY Seol et al., 2021)	-	Artificial intelligence-assisted clinical decision support for childhood asthma management: A randomized clinical trial	10.1371/journal.pone.0255261	electronic database	Seol	2021	PLoS One	Mayo Clinic, Minnesota, United States
No name (SM Overgaard et al., 2022)	-	A Technical Performance Study and Proposed Systematic and Comprehensive Evaluation of an ML-based CDS Solution for Pediatric Asthma	-	author	Overgaard	2022	AMIA Annual Symposium Proceedings Archive	Mayo Clinic, Rochester, Minnesota
No name (SM Overgaard et al., 2022)	-	Assessing socioeconomic bias in machine learning algorithms in health care: a case study of the HOUSES index	10.1093/jamia/ocac052	author	Juhn	2022	Journal of the American Medical Informatics Association	Mayo Clinic, Rochester, Minnesota
PULsE-AI (NR Hill et al., 2022)	-	Data Resource Profile: Clinical Practice Research Datalink (CPRD)	10.1093/ije/dyv098	electronic database	Emily Herrett	2015	International journal of epidemiology	London, UK
PULsE-AI (NR Hill et al., 2022)	-	Predicting atrial fibrillation in primary care using machine learning	10.1371/journal.pone.0224582	author	Nathan R. Hill	2019	PLoS One	Bristol-Myers Squibb Pharmaceutical Ltd, Uxbridge, UK

© 2024 Rakers MM et al. *JAMA Network Open*

Predictive ML algorithm	Product	Publication	doi	source	first author	year	source	center corresponding author
PULsE-AI (NR Hill et al., 2022)	-	Detecting undiagnosed atrial fibrillation in UK primary care: Validation of a machine learning prediction algorithm in a retrospective cohort study	10.1177/2047487320942338	author	Sara Sekelj	2020	Europen Journal of Preventive Cardiology	Imperial College Health Partners, London, UK
PULsE-AI (NR Hill et al., 2022)	-	Identification of undiagnosed atrial fibrillation using a machine learning risk prediction algorithm and diagnostic testing (PULsE-AI) in primary care: a multi-centre randomised controlled trial in England	10.1093/ehjdh/ztac009	electronic database	Nathan R. Hill	2022	European Heart Journal Digital Health	Bristol-Myers Squibb Pharmaceutical Ltd, Uxbridge, UK
No name (WE Herter et al., 2022)	-	Impact of a Machine Learning–Based Decision Support System for Urinary Tract Infections: Prospective Observational Study in 36 Primary Care Practices	10.2196/27795	electronic database	Herter	2022	JMIR Medical Informatics	Department of Public Health and Primary Care, Leiden University Medical Center, Leiden, Netherlands
No name (Y Wu et al., 2023)	-	Interpretable Machine Learning Models for Clinical Decision-Making in a High-Need, Value-Based Primary Care Setting	10.1056/CAT.21.0008	electronic database	Bhatt	2021	NEJM Catalyst Innovations in Care Delivery	Oak Street Health, Chicago, Illinois, USA
CUHAS-ROBUST (H Bumi et al., 2022)	-	Development and performance of CUHAS-ROBUST application for pulmonary rifampicin-resistance tuberculosis screening in Indonesia	10.1371/journal.pone.0249243	electronic database	Bumi Herman	2021	PLoS One	Chulalongkorn University, Bangkok, Thailand
CUHAS-ROBUST (H Bumi et al., 2022)	-	Artificial intelligence in overcoming rifampicin resistant-screening challenges in Indonesia: a qualitative study on the user experience of CUHAS-ROBUST	10.1108/JHR-11-2020-0535	electronic database	Bumi Herman	2021	Journal of Health Research	Chulalongkorn University, Bangkok, Thailand
No name (S Wnag et al., 2019)	-	Stepped-wedge randomised trial to evaluate population health intervention designed to increase appropriate anticoagulation in patients with atrial fibrillation	10.1136/bmjqs-2019-009367	electronic database	Wang	2022	BMJ Quality & Safety	Harvard Medical School, Boston, Massachusetts,

© 2024 Rakers MM et al. *JAMA Network Open*

Predictive ML algorithm	Product	Publication	doi	source	first author	year	source	center corresponding author
								USA
Firstderm (A Escalé-Besa et al., 2022) in primary care: feasibility study in clinical practice)	-	Evaluation of the Diagnostic Accuracy of an Online Artificial Intelligence Application for Skin Disease Diagnosis	10.2340/00015555-3624	author	Zaar	2020	Acta Dermato Venereologica	University of Gothenburg
Firstderm (A Escalé-Besa et al., 2022) in primary care: feasibility study in clinical practice)	-	Using artificial intelligence on dermatology conditions in Uganda: a case for diversity in training data sets for machine learning	10.4314/ahs.v23i2.86	author	Kamulegeya	2023	African Health Sciences	Sahlgrenska University Hospital
MEDO-Hip (JL Jeremko et al., 2023)		Traditional 510(k) Premarket Notification – MEDO Dx. - 510(k) Summary	-	Electronic database	Dornoosh Zonoobi	2020	Technical report	Singapore
MEDO-Hip (JL Jeremko et al., 2023)	-	Automated diagnosis of hip dysplasia from 3D ultrasound using artificial intelligence: A two-center multi-year study	10.1016/j.imu.2022.101082	electronic database	Ghasseminia	2022	Informatics in Medicine Unlocked	University of Alberta
MEDO-Hip (JL Jeremko et al., 2023)	-	Interobserver Variability of Hip Dysplasia Indices on Sweep Ultrasound for Novices, Experts, and Artificial Intelligence	10.1097/BPO.0000000000002065	electronic database	Ghasseminia	2022	Journal of Pediatric Orthopedics	University of Alberta
MEDO-Hip (JL Jeremko et al., 2023)	-	Remote diagnostic imaging using artificial intelligence for diagnosing hip dysplasia in infants: Results from a mixed-methods feasibility pilot study	10.1093/pch/pxad013	electronic database	Libon	2023	Paediatrics Child Health	University of Alberta
P3.AI (PH Chiang et al.,	-	Personalized Effect of Health Behavior on Blood Pressure: Machine Learning Based Prediction and	https://doi.org/10.1109/Healt	electronic	Chiang	2018	2018 IEEE 20th International	University of California at

© 2024 Rakers MM et al. *JAMA Network Open*

Predictive ML algorithm	Product	Publication	doi	source	first author	year	source	center corresponding author
2021)		Recommendation	hCom.2018.8531109	database			Conference on e-Health Networking, Applications and Services (Healthcom)	San Diego La Jolla
P3.AI (PH Chiang et al., 2021)	-	Offline and Online Learning Techniques for Personalized Blood Pressure Prediction and Health Behavior Recommendations	https://doi.org/10.1109/ACCESS.2019.2939218	electronic database	Chiang	2019	IEEE Access	University of California at San Diego La Jolla
P3.AI (PH Chiang et al., 2021)	-	Using Wearables and Machine Learning to Enable Personalized Lifestyle Recommendations to Improve Blood Pressure	https://doi.org/10.1109/JTEHM.2021.3098173	electronic database	Chiang	2021	IEEE Journal of Translational Engineering in Health and Medicine	University of California at San Diego La Jolla
P3.AI (PH Chiang et al., 2021)	-	An mHealth Lifestyle Intervention Service for Improving Blood Pressure using Machine Learning and IoMTs	https://doi.org/10.1109/ICDH55609.2022.00030	electronic database	Leitner	2022	2022 IEEE International Conference on Digital Health (ICDH)	University of California at San Diego La Jolla
EAGLE (X Yao et al., 2020)	-	Screening for cardiac contractile dysfunction using an artificial intelligence–enabled electrocardiogram	https://doi.org/10.1038/s41591-018-0240-2	electronic database	Attia	2019	Nature Medicine	Mayo Clinic, Rochester
EAGLE (X Yao et al., 2020)	-	Prospective validation of a deep learning ECG algorithm for the detection of left ventricular systolic dysfunction	https://doi.org/10.1111/jce.13889	electronic database	Attia	2019	Journal of Cardiovascular Electrophysiology	Mayo Clinic, Rochester
EAGLE (X Yao et al., 2020)	-	The Effects of Race and Ethnicity on a Deep Learning Model for ECG Analysis	https://doi.org/10.1161/CIRCEP.119.007988	electronic database	Noseworthy	2020	Circulation: Arrhythmia and Electrophysiology	Mayo Clinic, Rochester

Predictive ML algorithm	Product	Publication	doi	source	first author	year	source	center corresponding author
EAGLE (X Yao et al., 2020)	-	Artificial intelligence–enabled electrocardiograms for identification of patients with low ejection fraction: a pragmatic, randomized clinical trial	https://doi.org/10.1038/s41591-021-01335-4	electronic database	Yao	2021	Nature Medicine	Mayo Clinic, Rochester
EAGLE (X Yao et al., 2020)	-	Cost Effectiveness of an Electrocardiographic Deep Learning Algorithm to Detect Asymptomatic Left Ventricular Dysfunction	https://doi.org/10.1016/j.mayocp.2020.11.032	electronic database	Tseng	2021	Mayo Clinic Proceedings	Mayo Clinic, Rochester
EAGLE (X Yao et al., 2020)	-	Real-world performance, long-term efficacy, and absence of bias in the artificial intelligence enhanced electrocardiogram to detect left ventricular systolic dysfunction	https://doi.org/10.1093/ehjdh/ztac028	electronic database	Harmon	2022	European Heart Journal - Digital Health	Mayo Clinic, Rochester
EAGLE (X Yao et al., 2020)	-	Provider Perspectives on Artificial Intelligence–Guided Screening for Low Ejection Fraction in Primary Care: Qualitative Study	https://doi.org/10.2196/41940	electronic database	Barry	2022	JMIR AI	Mayo Clinic, Rochester
EAGLE (X Yao et al., 2020)	-	Clinician Adoption of an Artificial Intelligence Algorithm to Detect Left Ventricular Systolic Dysfunction in Primary Care.	https://doi.org/10.1016/j.mayocp.2022.04.008	electronic database	Rushlow	2022	Mayo Clinic Proceedings	Mayo Clinic, Rochester
EAGLE (X Yao et al., 2020)	-	Prospective evaluation of smartwatch-enabled detection of left ventricular dysfunction	https://doi.org/10.1038/s41591-022-02053-1	electronic database	Attia	2022	Nature Medicine	Mayo Clinic, Rochester
Medicine	Stethee pro	Heart Sound Segmentation Using Bidirectional LSTMs With Attention	10.1109/JBHI.2019.2949516	electronic database	Tharindu Fernando	2019	IEEE Journal of Biomedical and Health Informatics	Queensland University of Technology
Medicine	Stethee pro	Robust and Interpretable Temporal Convolution Network for Event Detection in Lung Sound Recordings	10.1109/JBHI.2022.3144314.	electronic database	Tharindu Fernando	2022	IEEE Journal of Biomedical and Health Informatics	Queensland University of Technology

© 2024 Rakers MM et al. *JAMA Network Open*

Predictive ML algorithm	Product	Publication	doi	source	first author	year	source	center corresponding author
Medicine	Stethee pro	Automatic segmentation and classification of cardiac cycles using deep learning and a wireless electronic stethoscope	10.1109/LSC. 2017.8268180	electronic database	Houman Ghaemmaghami	2017	IEEE Life Sciences Conference	Queensland University of Technology
Medicine	Stethee pro	Traditional 510(k) Premarket Notification - M3DICINE Pty Ltd. - 510(k) Summary	-	Electronic database	Ginger Cantor,	2020	Technical report	Australia
RootiMedical	Rooti Rx	Traditional 510(k) Premarket Notification Rooti Rx System ECG Event Recorder. - 510(k) Summary	-	Electronic database	Sue Chuang	2016	Technical report	Taipei
RootiMedical	Rooti Rx	The quality of ECG data acquisition, and diagnostic performance of a novel adhesive patch for ambulatory cardiac rhythm monitoring in arrhythmia detection	10.1016/j.jelectrocard.2019.02.012	electronic database	M. Remzi Karaoguz	2019	Journal of Electrocardiology	Koç University Hospital, Istanbul
RootiMedical	Rooti Rx	Screening of obstructive sleep apnea in patients who snore using a patch-type device with electrocardiogram and 3-axis accelerometer	10.5664/jcsm. 8462#d1e401	electronic database	Ying-Shuo Hsu	2020	Journal of Clinical Sleep Medicine	Shin Kong Wu-Ho-Su Memorial Hospital
DigitalDiagnostics	ID-Dx	Improved Automated Detection of Diabetic Retinopathy on a Publicly Available Dataset Through Integration of Deep Learning	10.1167/iovs.16-19964	website	M. Abramoff	2016	Investigate Ophthalmology & Visual science	University of lowa
DigitalDiagnostics	ID-Dx	Validation of automated screening for referable diabetic retinopathy with the IDx-DR device in the Hoorn Diabetes Care System	10.1111%2Faos.13613	website	A. van der Heijden	2017	Acta Ophthalmologica	VUMC, The Netherlands
DigitalDiagnostics	ID-Dx	Pivotal trial of an autonomous AI-based diagnostic system for detection of diabetic retinopathy in primary care offices	10.1038/s41746-018-0040-6	website	M. Abramoff	2018	Nature	University of lowa
DigitalDiagnostics	ID-Dx	Traditional 510(k) Premarket Notification – Idx-DR - 510(k) Summary	https://www.accessdata.fda.gov/cdrh_docs/reviews/DEN18	Electronic database	M. Abramoff	2018	Technical report	-

Predictive ML algorithm	Product	Publication	doi	source	first author	year	source	center corresponding author
			0001.pdf					
DigitalDiagnostics	ID-Dx	Diagnostic Accuracy of a Device for the Automated Detection of Diabetic Retinopathy in a Primary Care Setting	10.2337/dc18-0148	website	F. Verbraak	2019	American Diabetes Association	VUMC, The Netherlands
DigitalDiagnostics	ID-Dx	A pilot autonomous AI-based DR screening in Poland	10.1111/j.1755-3768.2019.5078	website	Andrezej Grzybowski	2019	Acta Ophthalmologica	University of Warmia and Mazury, Poland
DigitalDiagnostics	ID-Dx	A Framework to Evaluate Ethical Considerations with ML-HCA Applications—Valuable, Even Necessary, but Never Comprehensive	10.1080/15265161.2020.1827695	website	Danton Char	2020	The American Journal of Bioethics	Standford University School of Medicine
DigitalDiagnostics	ID-Dx	Automated and Computer-Assisted Detection, Classification, and Diagnosis of Diabetic Retinopathy	10.1089/tmj.2020.0008	website	M. Abramoff	2020	American Telemedicine Association	University of lowa
DigitalDiagnostics	ID-Dx	Practice Guidelines for Ocular Telehealth-Diabetic Retinopathy, Third Edition	10.1089%2Ftmj.2020.0006	website	M. Horton	2020	American Telemedicine Association	Phoenix, Arizona
DIgitalDiagnostics	ID-Dx	Validation of Automated Screening for Referable Diabetic Retinopathy With an Autonomous Diagnostic Artificial Intelligence System in a Spanish Population	10.1177/1932296820906212	website	Abhay Shah	2020	Journal of Diabetes Science and Technology	IA, USA
DIgitalDiagnostics	ID-Dx	Identifying Ethical Considerations for Machine Learning Healthcare Applications	10.1080/15265161.2020.1819469	author	Danton Char	2020	THe American Journal of Bioethics	Standford University School of Medicine
DIgitalDiagnostics	ID-Dx	Lessons Learned About Autonomous AI: Finding a Safe, Efficacious, and Ethical Path Through the Development Process	10.1016/j.ajo.2020.02.022	website	M. Abramoff	2020	American Journal of Opthalmology	University of lowa

© 2024 Rakers MM et al. *JAMA Network Open*

Predictive ML algorithm	Product	Publication	doi	source	first author	year	source	center corresponding author
DigitalDiagnostics	ID-Dx	Cost-effectiveness of Autonomous Point-of-Care Diabetic Retinopathy Screening for Pediatric Patients With Diabetes	10.1001/jamaophthalmol.2020.3190	author	Risa M. Wolf	2020	JAMA Ophtalmolgy	Johns Hopkins School of Medicine, Baltimore
DIgitalDiagnostics	ID-Dx	Autonomous Artificial Intelligence in Diabetic Retinopathy: From Algorithm to Clinical Application	10.1177/1932296820909900	website	Roomasa Channa	2021	Journal of Diabetes Science and Technology	Houston, TX, USA
DigitalDiagnostics	ID-Dx	Foundational Considerations for Artificial Intelligence Using Ophthalmic Images	10.1016/j.ophtha.2021.08.023	website	M. Abramoff	2021	American Journal of Opthalmology	University of lowa
DigitalDiagnostics	ID-Dx	A reimbursement framework for Artificial Intelligence in Healthcare	10.1038/s41746-022-00621-w	author	M. Abramoff	2022	Nature Digital Medicine, in press	University of Iowa
DigitalDiagnostics	ID-Dx	Lessons Learned About Autonomous AI: Finding a Safe, Efficacious, and Ethical Path Through the Development Process	10.1016/j.ajo.2020.02.022	author	M. Abramoff	2022	Article in press	University of Iowa
Qompium NV	FibriCheck	Validation of a smartphone based photoplethysmographic beat detection algorithm for normal and ectopic complexes	Conference	website	L. Drijkoningen	2014	Computing in Cardiology	Hasselt University, Belgium
Qompium NV	FibriCheck	Clinical Validation of Heart Rate Apps: Mixed-Methods Evaluation Study	10.2196/mhealth.7254	website	T. Vandenberk	2017	JMIR mHealth and uHealth	Ziekenhuis Oost-Limburg, Belgium
Qompium NV	FibriCheck	Traditional 510(k) Premarket Notification – Idx-DR - 510(k) Summary	https://www.accessdata.fda.gov/cdrh_docs/pdf17/K173872.pdf	Electronic database	Patsy Trisler	2018	Technical report	Maryland
Qompium NV	FibriCheck	Performance of an artificial intelligence algorithm to detect atrial fibrillation on a 24-hour continuous photoplethysmography recording using a	https://doi.org/10.1093/eurheartj/ehab724.0	website	H. Gruwez	2021	European Heart Journal	University of Leuven, Belgium

© 2024 Rakers MM et al. *JAMA Network Open*

Predictive ML algorithm	Product	Publication	doi	source	first author	year	source	center corresponding author
		smartwatch: ACURATE study	489					
Cardio-Phoenix	Cardio-HART	Unraveling diagnostic co-morbidity makeup of each HF category as 2 characteristically derived by ECG- and ECHO-findings, a 3 prevalence analysis	10.1101/2021.09.30.21264236v1	electronic database	Azfar Zaman	2021	BMJ Yale	Newcastle University
CSD Labs GmbH	eMurmur	REDUCING INAPPROPRIATE USE OF ECHOCARDIOGRAPHY IN CHILDREN: SCREENING HEART MURMURS WITH AN AI WITH ENABLED STETHOSCOPE	https://hvpaa.org/conference/	website	Christine Kuryla	2019	Conference	Johns Hopkins School of Medicine, Baltimore
CSD Labs GmbH	eMurmur	Computerized Automatic Diagnosis of Innocent and Pathologic Murmurs in Pediatrics: A Pilot Study	10.1111/chd.12328	website	Lilian S W Lai	2016	Congenital Heart Disease	Ottawa, Canada
CSD Labs GmbH	eMurmur	Artificial Intelligence-Assisted Auscultation of Heart Murmurs: Validation by Virtual Clinical Trial	10.1007/s00246-018-2036-z	website	W. Reid Thompson	2018	Pediatric Cardiology	Johns Hopkins Children's Center
CSD Labs GmbH	eMurmur	IMPLEMENTATION AND UTILITY OF A PHONE-BASED APPLICATION FOR TEACHING AUSCULTATION SKILLS TO INCOMING PEDIATRIC CARDIOLOGY FELLOWS	10.1016/j.acap.2020.06.070	website	Inger Olson	2020	Academic Pediatrics	Stanford University, USA
CSD Labs GmbH	eMurmur	Use of Telemedicine with An Artificial Intelligence-Enabled Stethoscope to Support Decision-Making and Reduce Inappropriate Use of Echocardiography in Children with Heart Murmurs	10.1542/peds.147.3MA10.989a	website	W. Reid Thompson	2021	Pediatrics (abstract)	Johns Hopkins Children's Center
CSD Labs GmbH	eMurmur	Feasibility of using an artificial intelligence-enabled stethoscope and telemedicine to improve referrals and reduce inappropriate use of echocardiography in children with heart murmurs	Conference	website	Christine Kuryla	2021	Conference	Johns Hopkins School of Medicine, Baltimore

© 2024 Rakers MM et al. *JAMA Network Open*

Predictive ML algorithm	Product	Publication	doi	source	first author	year	source	center corresponding author
CSD Labs GmbH	eMurmur	Feasibility assessment of AI-assisted home-based remote auscultation to optimize echocardiogram referrals	Poster	website	Jessica Campanile	2021	Poster presentation	Johns Hopkins School of Medicine, Baltimore
CSD Labs GmbH	eMurmur	Traditional 510(k) Premarket Notification – Idx-DR - 510(k) Summary	https://www.accessdata.fda.gov/cdrh_docs/pdf22/K220766.pdf	Electronic database	Allison Komiyama	2022	Technical report	San Diego, California
Hefei Mintti Medical Technology Co. Ltd	Smartho-D2 Electronic Stethoscope	Cardiac Sound Localization and Identification Method for Electronic Stethoscope	10.1109/ICSPCC.2018.8567742	author	Ning Zhou	2018	IEEE	Soochow University, China
Hefei Mintti Medical Technology Co. Ltd	Smartho-D2 Electronic Stethoscope	Detection of Adventitious Respiratory Sounds based on Convolutional Neural Network	10.1109/ICIIBMS46890.2019.8991459	author	Renyu Liu	2019	IEEE	Soochow University, China
Hefei Mintti Medical Technology Co. Ltd	Smartho-D2 Electronic Stethoscope	The Automatic Repairing Method Addressing Clipping Distortions and Frictional Noises in Electronic Stethoscope	10.1109/ICBCB.2019.8854669	author	Ning Zhou	2019	IEEE	Soochow University, China
Hefei Mintti Medical Technology Co. Ltd	Smartho-D2 Electronic Stethosc	Traditional 510(k) Premarket Notification – Electronic Stehoscope – smartho-D2 - 510(k) Summary	https://www.accessdata.fda.gov/cdrh_docs/pdf19/K191667.pdf	Electronic database	Shengshen Cai	2019	Technical report	

© 2024 Rakers MM et al. *JAMA Network Open*

Predictive ML algorithm	Product	Publication	doi	source	first author	year	source	center corresponding author
	ope							
Hefei Mintti Medical Technology Co. Ltd	Smartho-D2 Electronic Stethoscope	Machine-LearningLearning-based Aortic Stenosis Detection for Electronic Stethoscope	10.1109/WCSP52459.2021.9613207	author	Zhen Shi	2021	IEEE	Soochow University, China
Eko Devices Inc.	EKO Analysis Software	Real-World Evaluation of the Eko Electronic Teleauscultation System	10.1007/s00246-018-1972-y	website	Shashank Behere	2018	Pediatric Cardiology	Hospital for Children, Wilmington, USA
Eko Devices Inc.	EKO Analysis Software	Prospective Analysis of Utility of Signals From an Ecg-Enabled Stethoscope to Automatically Detect a Low Ejection Fraction Using Neural Network Techniques Trained From the Standard 12-Lead Ecg	Abstract	website	Z.I. Attia	2019	Circulation	Mayo Clinic, USA
Eko Devices Inc.	EKO Analysis Software	Screening for cardiac contractile dysfunction using an artificial intelligence–enabled electrocardiogram	10.1038/s41591-018-0240-2	website	Z.I. Attia	2019	Nature Medicine	Mayo Clinic, USA
Eko Devices Inc.	EKO Analysis Software	Deep Learning Algorithm for Automated Cardiac Murmur Detection via a Digital Stethoscope Platform	10.1161/JAHA.120.019905	website	John S Chorba	2021	Journal of the American Heart Association	University of California, USA
Eko Devices Inc.	EKO Analysis Software	Point-of-care screening for heart failure with reduced ejection fraction using artificial intelligence during ECG-enabled stethoscope examination in London, UK: a prospective, observational, multicentre study	10.1016/S2589-7500(21)00256-9	website	Patrik Bachtiger	2022	The Lancet Digital Health	Imperial College London, London, UK

Predictive ML algorithm	Product	Publication	doi	source	first author	year	source	center corresponding author
Eko Devices Inc.	EKO Analysis Software	Automated Detection of Low Ejection Fraction from a One-lead Electrocardiogram: Application of an AI algorithm to an ECG-enabled Digital Stethoscope	10.1093/ehjdh/ztac030	website	Z.I. Attia	2022	European Heart Journal Digital Health	Mayo Clinic, USA
Eko Devices Inc.	EKO Analysis Software	Traditional 510(k) Premarket Notification – CORE 500 Digital Stehoscope - 510(k) Summary	accessdata.fda.gov/cdrh_docs/pdf23/K230111.pdf	Electronic database	Sam Huang	2023	Technical report	Oakland, California
KOSMOS	EchoNous	Traditional 510(k) Premarket Notification – Kosmos- 510(k) Summary	accessdata.fda.gov/cdrh_docs/pdf21/K212100.pdf	Electronic database	Prithul Bom	2021	Technical report	Saint Paul
KOSMOS	EchoNous	Clinical validation of an artificial intelligence-assisted algorithm for automated quantification of left ventricular ejection fraction in real time by a novel handheld ultrasound device	10.1093/ehjdh/ztac001	lectronic database	Stella-Lida Papadopoulou	2022	European Heart Journal	Papageorgiou General Hospital (Greece)
Eyenuk Inc.	EyeArt	Development and Validation of a Deep Learning Algorithm for Detection of Diabetic Retinopathy in Retinal Fundus Photographs	10.1001/jama.2016.17216	electronic database	Varun Gulshan	2016	Jama Network Open	Eyenuk California
Eyenuk Inc.	EyeArt	Automated Diabetic Retinopathy Image Assessment Software: Diagnostic Accuracy and Cost-Effectiveness Compared with Human Graders	10.1016/j.ophtha.2016.11.014	electronic database	Adnan Tufail	2016	Ophthalmology	Moorfields Eye Hospital, London, UK
Eyenuk Inc.	EyeArt	An observational study to assess if automated diabetic retinopathy image assessment software can replace one or more steps of manual imaging grading and to determine their cost-effectiveness.	10.3310/hta20920	electronic database	Tufail A.	2016	Health Technology Assessment	University College London Institute of Ophthalmology.

© 2024 Rakers MM et al. *JAMA Network Open*

**Predictive ML algorithm**	**Product**	**Publication**	**doi**	**source**	**first author**	**year**	**source**	**center corresponding author**
Eyenuk Inc.	EyeArt	The Value of Automated Diabetic Retinopathy Screening with the EyeArt System: A Study of More Than 100,000 Consecutive Encounters from People with Diabetes	10.1089%2Fdia.2019.0164	electronic database	Malavika Bhaskaranand	2019	Diabetes Technology & Therapeutics	Eyenuk California
Eyenuk Inc.	EyeArt	Performance of a Deep-Learning Algorithm vs Manual Grading for Detecting Diabetic Retinopathy in India	10.1001/jamaophthalmol.2019.2004	electronic database	Varun Gulshan	2019	JAMA Ophthalmology	Google Inc, Mountain View, California
Eyenuk Inc.	EyeArt	Traditional 510(k) Premarket Notification – EyeArt- 510(k) Summary	accessdata.fda.gov/cdrh_docs/pdf20/K200667.pdf	Electronic database	Kaushal Solanki	2020	Technical report	Los Angeles, California
Eyenuk Inc.	EyeArt	Pivotal Evaluation of an Artificial Intelligence System for Autonomous Detection of Referrable and Vision-Threatening Diabetic Retinopathy	10.1001/jamanetworkopen.2021.34254	website	Eli Ipp	2021	JAMA Network Open	Eyenuk California
Eyenuk Inc.	EyeArt	Diabetic Retinopathy Screening with Automated Retinal Image Analysis in a Primary Care Setting Improves Adherence to Ophthalmic Care	10.1016/j.oret.2020.06.016	electronic database	James Liu	2022	Ophthalmology Retina	Washington University School of Medicine, USA
Coala Life AB	Coala Heart Monitor	Symptom-Ruled Real World Arrhythmic Recordings With an Internet Based System	Abstract	electronic database	Olsson	2018	Circulation	Sturebadet Health Care, Stockholm, Sweden
Coala Life AB	Coala Heart Monitor	Performance evaluation of dual vs. single lead automatic, real-world arrhythmic ECG recordings.	Conference	electronic database	Olsson	2019	Abstract, conference	Sturebadet Health Care, Stockholm, Sweden
Coala Life AB	Coala Heart Monitor	Traditional 510(k) Premarket Notification – Coala Heart Monitor- 510(k) Summary	accessdata.fda.gov/cdrh_docs/pdf18/K182040.pdf	Electronic database	Piere Bounaud	2019	Technical report	San Diego, California

© 2024 Rakers MM et al. *JAMA Network Open*

**Predictive ML algorithm**	**Product**	**Publication**	**doi**	**source**	**first author**	**year**	**source**	**center corresponding author**
Coala Life AB	Coala Heart Monitor	Evaluation of an enhanced, cloud-based AF-detection algorithm based on real-world arrhythmic	Conference	electronic database	Olsson	2019	Abstract, conference	Sturebadet Health Care, Stockholm, Sweden
Coala Life AB	Coala Heart Monitor	Instant analysis of the ECG with a new digital technique during palpitations reduces symptoms, anxiety, depression, and increases HRQOL in women	Conference	electronic database	Carnlof	2019	Abstract, conference	Sturebadet Health Care, Stockholm, Sweden
Coala Life AB	Coala Heart Monitor	Symptomatic palpitations causing anxiety in women, what are the underlying arrhythmias?	Conference	electronic database	Per Insulander	2019	Abstract, conference	Institutet and Karolinska University Hospital, Stockholm, Sweden
Coala Life AB	Coala Heart Monitor	Device profile of the Coala Heart Monitor for remote monitoring of the heart rhythm: overview of its efficacy	10.1080/17434440.2020.1732814	electronic database	Per Insulander	2020	Expert Review of Medical Devices	Institutet and Karolinska University Hospital, Stockholm, Sweden
Babylon Health Ltd	Babylon	A Comparison of Artificial Intelligence and Human Doctors for the Purpose of Triage and Diagnosis	10.3389/frai.2020.543405	website	Adam Baker	2020	Frontiers in Artificial Intelligence	Babylon Health
Babylon Health Ltd	Babylon	Improving the accuracy of medical diagnosis with causal machine learning	10.1038/s41467-020-17419-	website	Jonathan G. Richens	2020	Nature Communications	Babylon Health
Huma Therapeutics	Huma	Accelerometer-derived sleep onset timing and cardiovascular disease incidence: a UK Biobank cohort study	10.1093/ehjdh/ztab088	website	Shahram Nikbakhtian	2021	European Heart Journal Digital Health	Huma Therapeutics

© 2024 Rakers MM et al. *JAMA Network Open*

**Predictive ML algorithm**	**Product**	**Publication**	**doi**	**source**	**first author**	**year**	**source**	**center corresponding author**
Huma Therapeutics	Huma	Development and Validation of Risk Scores for All-Cause Mortality for a Smartphone-Based “General Health Score” App: Prospective Cohort Study Using the UK Biobank	10.2196/25655	website	Ashley K Clift	2021	JMIR Mhealth Uhealth	Imperial College London
Huma Therapeutics	Huma	Development of an accessible 10-year Digital CArdioVAscular (DiCAVA) risk assessment: a UK Biobank study	10.1093/ehjdh/ztab057	website	Nikola Dolezalova	2021	European Heart Journal Digital Health	Huma Therapeutics
Huma Therapeutics	Huma	Implementation of a mHealth solution to remotely monitor patients on a cardiac surgical waiting list: service evaluation	10.1093/jamiaopen/ooab053	website	Bernard Dillon Obika	2021	JAMIA Open	Huma Therapeutics
Huma Therapeutics	Huma	Development of Digitally Obtainable 10-Year Risk Scores for Depression and Anxiety in the General Population	10.3389/fpsyt.2021.689026	website	Davide Morelli	2021	Frontiers in Psychiatry	Huma Therapeutics
Huma Therapeutics	Huma	Traditional 510(k) Premarket Notification – Huma RPM- 510(k) Summary	accessdata.fda.gov/cdrh_docs/pdf23/K230214.pdf	Electronic database	Mani Shanmugham	2023	Technical report	New York
Skin Analytics Limited	DERM	Detection of Malignant Melanoma Using Artificial Intelligence: An Observational Study of Diagnostic Accuracy	10.5826/dpc.1001a11	website	Michael Phillips	2019	Dermatology Practical & Conceptual	Royal Perth Hospital
Skin Analytics Limited	DERM	Assessment of Accuracy of an Artificial Intelligence Algorithm to Detect Melanoma in Images of Skin Lesions	10.1001/jamanetworkopen.2019.13436	website	Michael Phillips	2019	JAMA Network Open	Harry Perkins Institute of Medical Research (Perth)
ResApp	ResApp Health	Cough Sound Analysis Can Rapidly Diagnose Childhood Pneumonia	10.1007/s10439-013-0836-0	website	Udantha R Abeyratne	2013	Annals of Biomedical Engineering	University of Queensland

© 2024 Rakers MM et al. *JAMA Network Open*

Predictive ML algorithm	Product	Publication	doi	source	first author	year	source	center corresponding author
ResApp	ResApp Health	Automatic Identification of Wet and Dry Cough in Pediatric Patients with Respiratory Diseases	10.1007/s10439-013-0741-6	website	Vinayak Swarnkar	2013	Annals of Biomedical Engineering	University of Queensland
ResApp	ResApp Health	Automatic cough segmentation from non-contact sound recordings in pediatric wards	10.1016/j.bspc.2015.05.001	website	Yusuf A Amrulloh	2015	Biomedical Signal Processing and Control	University of Queensland
ResApp	ResApp Health	Wavelet Augmented Cough Analysis for Rapid Childhood Pneumonia Diagnosis	10.1109/TBME.2014.2381214	website	Keegan Kosasih	2015	IEEE Transactions on Biomedical Engineering	University of Queensland
ResApp	ResApp Health	Exhaustive mathematical analysis of simple clinical measurements for childhood pneumonia diagnosis	10.1007/s12519-017-0019-4	website	Keegan Kosasih	2017	World Journal of Pediatrics	University of Queensland
ResApp	ResApp Health	Automatic Croup Diagnosis Using Cough Sound Recognition	10.1109/TBME.2018.2849502	website	Roneel V Sharan	2019	IEEE Transactions on Biomedical Engineering	University of Queensland
ResApp	ResApp Health	A prospective multicentre study testing the diagnostic accuracy of an automated cough sound centred analytic system for the identification of common respiratory disorders in children	10.1186/s12931-019-1046-6	website	Paul Porter	2019	Respiratory Research	Curtin University
ResApp	ResApp Health	Diagnosing Chronic Obstructive Airway Disease on a Smartphone Using Patient-Reported Symptoms and Cough Analysis: Diagnostic Accuracy Study	10.2196/24587	website	Paul Porter	2020	JMIR Formative Research	Curtin University

© 2024 Rakers MM et al. *JAMA Network Open*

Predictive ML algorithm	Product	Publication	doi	source	first author	year	source	center corresponding author
ResApp	ResApp Health	Diagnosing community-acquired pneumonia via a smartphone-based algorithm: a prospective cohort study in primary and acute-care consultations	10.3399/BJGP.2020.0750	website	Paul Porter	2021	British Journal of General Practice	Curtin University
ResApp	ResApp Health	Identifying acute exacerbations of chronic obstructive pulmonary disease using patient-reported symptoms and cough feature analysis	10.1038/s41746-021-00472-x	website	Scott Claxton	2021	npj Digital Medicine	Joondalup Health Campus
ResApp	ResApp Health	Traditional 510(k) Premarket Notification – SleepCheck Rx - 510(k) Summary	accessdata.fda.gov/cdrh_docs/pdf21/K213360.pdf	Electronic database	Neroli Anderson	2022	Technical report	Brisbane Queensland Australia
MobileODT	AVEC	Remote quality assurance in cervical cancer screening in low resource settings using a handheld smartphone-based colposcope	10.1117/12.2086377	website	Christophe Millien	2015	Proceedings of SPIE	Hôpital Universitaire de Mirebalais (Haiti)
MobileODT	AVEC	Mobile colposcopy in urban and underserved suburban areas in Baja California	10.1117/12.2218697	website	Marta Madiedo	2016	Proceedings of SPIE	Fronteras Unidas Pro-Salud (Mexico)
MobileODT	AVEC	MobileODT: a case study of a novel approach to an mHealth-based model of sustainable impact	10.21037/mhealth.2016.03.10	website	Jonah Mink	2016	mHealth	MobileODT
MobileODT	AVEC	Real-Time Monitoring and Evaluation of a Visual-Based Cervical Cancer Screening Program Using a Decision Support Job Aid	10.3390/diagnostics6020020	website	Curtis W Peterson	2016	diagnostics	MobileODT
MobileODT	AVEC	Measurement and evaluation of digital cervicography programs in two cervical cancer screening camps in East Africa	10.1117/12.2252249	website	Curtis W Peterson	2017	Proceedings of SPIE	MobileODT
MobileODT	AVEC	Cloud-based processing of multi- spectral imaging data	10.1117/12.2252189	website	Amir S Bernat	2017	Proceedings of SPIE	MobileODT
MobileODT	AVEC	Characterization of cervigram image sharpness using multiple self- referenced measurements and	10.1117/12.2292179	website	Mayoore Jaiswal	2018	Proceedings of SPIE	Intellectual Ventures

© 2024 Rakers MM et al. *JAMA Network Open*

Predictive ML algorithm	Product	Publication	doi	source	first author	year	source	center corresponding author
		random forest classifiers						Laboratory
MobileODT	AVEC	Portable, low-cost multispectral imaging system: design, development, validation, and utilization	10.1117/1.JBO.23.12.121612	website	Frank J Bolton	2018	Journal of Biomedical Optics	MobileODT
MobileODT	AVEC	Low-Cost Instructional Apparatus to Improve Training for Cervical Cancer Screening and Prevention	10.1097/AOG.0000000000003140	website	Sonia Parra	2019	Obstetrics & Gynecology	Rice University (Houston)
MobileODT	AVEC	Prospective cohort study examining cervical cancer screening methods in HIV-positive and HIV-negative Cambodian Women: a comparison of human papilloma virus testing, visualization with acetic acid and digital colposcopy	10.1136/bmjopen-2018-026887	website	Sovannara Thay	2019	Obstetrics & Gynecology	Sihanouk Hospital Center of Hope (Cambodia)
MobileODT	AVEC	Introduction of Mobile Colposcopy as a Primary Screening Tool for Different Socioeconomic Populations in Urban India	-	website	Renuka Matti	2019	Pan Asian Journal of Obstetrics & Gynecology	Dr LH Hiranandani Hospital (Mumbai)
SkinVision	SkinVision	Real-time acquisition of quality verified nonstandardized color images for skin lesions risk assessment — A preliminary study	10.1109/ICSTCC.2014.6982415	author	A Udrea	2014	18th International Conference on System Theory, Control and Computing (ICSTCC)	University Politehnica of Bucharest, SkinVision
SkinVision	SkinVision	Accuracy of a smartphone application using fractal image analysis of pigmented moles compared to clinical diagnosis and histological result	10.1111/jdv.12648	website	T Maier	2014	Journal of the European Academy of Dermatology and Venerealogy	University Hospital of Munich

© 2024 Rakers MM et al. *JAMA Network Open*

Predictive ML algorithm	Product	Publication	doi	source	first author	year	source	center corresponding author
SkinVision	SkinVisi on	mHealth App for Risk Assessment of Pigmented and Nonpigmented Skin Lesions-A Study on Sensitivity and Specificity in Detecting Malignancy	10.1089/tmj.2 016.0259	website	Monique Thissen	2017	Telemedicine Journal and E-Health	Catharina Hospital (Eindhoven), MUMC+, Maastricht University
SkinVision	SkinVisi on	Development of Smartphone Apps for Skin Cancer Risk Assessment: Progress and Promise	10.2196/13376	website	Tiago M de Carvalho	2019	JMIR Dermatology	Erasmus Medical Center (Rotterdam)
SkinVision	SkinVisi on	Accuracy of a smartphone application for triage of skin lesions based on machine learning algorithms	10.1111/jdv.15 935	website	A Udrea	2020	Journal of the European Academy of Dermatology and Venerealogy	University Politehnica of Bucharest, SkinVision
SkinVision	SkinVisi on	Validation of a Market-Approved Artificial Intelligence Mobile Health App for Skin Cancer Screening: A Prospective Multicenter Diagnostic Accuracy Study	10.1159/00052 0474	electroni c database	Sangers T	2022	Dermatology	Erasmus Medical Center (Rotterdam)
SkinVision	SkinVisi on	Views on mobile health apps for skin cancer screening in the general population: an in-depth qualitative exploration of perceived barriers and facilitators.	10.1111/bjd.20 441	electroni c database	Sangers T	2021	Dermatology	Erasmus Medical Center (Rotterdam)
SkinVision	SkinVisi on	FDA Executive Summary Reclassification Panel Meeting on Skin Lesion Analyzers	fda.gov/media/ 160252/downl oad	Electron ic database	-	2022	Technical report / meeting	-
SkinVision	SkinVisi on	Artificial intelligence in mobile health for skin cancer diagnostics at home (AIM HIGH): a pilot feasibility study	10.1016/j.eclin m.2023.10201 9	electroni c database	A Smak Gregoor	2023	eClinicalMedici ne Lancet	Erasmus Medical Center (Rotterdam)
Minuteful - kidney test	Healthy. io Ltd.	Traditional 510(k) Premarket Notification – Minuteful – kidney test - 510(k) Summary	accessdata.fda. gov/cdrh_docs	Electron ic	Ron Zohar	2022	Technical report	Tel Aviv, Israel

© 2024 Rakers MM et al. *JAMA Network Open*

Predictive ML algorithm	Product	Publication	doi	source	first author	year	source	center corresponding author
			/pdf21/K210069.pdf	database				
Minuteful - kidney test	Healthy.io Ltd.	Exploring Implementation of a Home-Based Test for Kidney Disease: A Feasibility Study	-	electronic database	J. Gregoire	2023	AMGA	Valley Medical Group
Minuteful - kidney test	Healthy.io Ltd.	Evaluating the feasibility and acceptability of home-based urinalysis for albumin-creatinine ratio with smartphone technology: A quality improvement project	10.1111/jorc.12460	electronic database	N Thomas	2023	Journal of Renal Care	London South Bank University
Medicalgorithmics	DeepRhythmAI	Traditional 510(k) Premarket Notification - DeepRhythmAI - 510(k) Summary	-	electronic database	-	2022	Technical report	-
IRNF App	Irregular Ryhthm Notification Feature (IRNF) 2.0 App	Traditional 510(k) Premarket Notification - Irregular Ryhthm Notification Feature (IRNF) 2.0 App - 510(k) Summary	-	electronic database	-	2021	Technical report	-
IRNF App	Irregular Ryhthm Notification Feature (IRNF) 2.0 App	Atrial Fibrillation Algorithms Clinical Validation Study	-	electronic database	-	2022	Technical report	-
IRNF App	Irregular Ryhthm Notification Feature	Atrial Fibrillation Algorithms Clinical Validation Study - Study Protocol and Statistical Analysis Plan	-	electronic database	-	2021	Technical report	-

© 2024 Rakers MM et al. *JAMA Network Open*

**Predictive ML algorithm**	**Product**	**Publication**	**doi**	**source**	**first author**	**year**	**source**	**center corresponding author**
	(IRNF) 2.0 App							

© 2024 Rakers MM et al. *JAMA Network Open*

eTable 3 – Overview of the availability of evidence per predictive ML algorithm

Evidence for each predictive ML algorithm was assessed according to Dutch AIPA guideline requirements per life cycle phase (Table 1, Box 2, and Appendix 4), utilizing a color-coded system (green for complete, yellow for partial, grey for none, and orange for predictive ML algorithms not yet in phase 6), with opaque colours used if authors or owners withheld non-public information. Green, yellow, and grey/orange were assigned values of 2, 1, and 0, respectively,

Name of AIPA	Responded and provided	Phase 1: Preparation and		Phase 2: Development of the AI-model								Phase 3: Validation of the AI-model					
		Datamanagementplan		Definement target use		Analysis and modeling steps	Internal validation	Robustness	Size of the dataset for AI-model development	Reproducibility and replicability		Evaluation of (statisical) characteristics of the AI-model		Fairness and algoritmic bias	Determining the outcome variable	Reproducibility and replicability	Size of the dataset for external validation
**Included studies from literature review**																	
Aifred	x																
VisualDx																	
DSS																	
ECG-																	
AI																	
Automate																	
Medicatio																	
AI for risk																	
A-GPS	x																
PULsE-AI	x																
ML-based																	
Risk																	
CUHAS-																	
Risk-																	
Personalis																	
AI-ECG																	
MEDO-Hip																	
Firstderm	x																
**Commercially available CE- and FDA-marked AI tools**																	
Tyto																	
Peerbridge																	
Rooti Rx																	
IDx-DR	x																
Fibricheck																	
Cardio-	x																
eMurmur	x																
Smartho-	x																
Biosticker																	
EKO																	
KOSMOS																	
EyeArt																	
Coala																	
MyAsthma																	
Babylon																	
MedoPad/																	
DERM																	
ResAppDx-																	
AVEC																	
Kata																	
SkinVision	x																
DeepRhythmAI																	
IRNF App																	
Minuteful - kidney test																	
Zeus																	

Phase 4: Development of the necessary software application			Phase 5: Impact assessment of the AI-model in combination with the software						Phase 6: Implementation and use of the AI-model with software in daily practice				
Explainability, transparency, design and information	Required standards and regulations		Impact assessment				Health Technology Assessment (HTA)		Implementationplan	Monitoring	Education		**Average score**
													18/42
													3/42
													16/42
													27/42
													13/48
													35/48
													4/42
													4/42
													26/48
													36/48
													18/48
													35/48
													26/42
													0/42
													26/42
													37/42
													19/48
													24/42
													4/48
													4/48
													20-48
													22/48
													28/48
													14/48
													28/48
													10/48
													4/48
													27/48
													11/48
													35/48
													4/48
													4/48
													13/48
													13/48
													15/48
													22/48
													8/48
													4/48
													25/48
													5/48
													20/48
													8/48
													8/48

forming availability scores.

Green indicates the publicy availability of evidence; solid green indicates the evidence is publicly available, and opaque green indicates that authors or owners indicated that information regarding the requirements was restricted. Orange indicates the evidence available partially covers the requirement; solid orange indicates the evidence is publicly available, and opaque yellow indicates that authors or owners indicated that information regarding the requirements was restricted.
