# Supplementary material for: Availability of Evidence for Predictive Machine Learning Algorithms in Primary Care: A Systematic Review
Source: JAMA Netw Open. 2024 Sep 12;7(9):e2432990. doi: 10.1001/jamanetworkopen.2024.32990 (PMC11393722; doi:10.1001/jamanetworkopen.2024.32990)
Supplement: Supplement 2. — Data Sharing Statement [file jamanetwopen-e2432990-s002.pdf]

Data Sharing Statement

Rakers. Availability of Evidence for Predictive Machine Learning Algorithms in Primary Care. *JAMA Netw Open*. Published September 12, 2024. doi:10.1001/jamanetworkopen.2024.32990

Data
Data available: Yes
Data types: Data (not involving human participants)
How to access data: Data requests can be send to m.m.rakers@lumc.nl
When available: With publication

Supporting Documents
Document types: None

Additional Information
Who can access the data: Anyone requesting the data
Types of analyses: Any purpose
Mechanisms of data availability: With investigator support
